# Nkx2.1 regulates the generation of telencephalic astrocytes during embryonic development

**DOI:** 10.1038/srep43093

**Published:** 2017-03-07

**Authors:** Shilpi Minocha, Delphine Valloton, Yvan Arsenijevic, Jean-René Cardinaux, Raffaella Guidi, Jean-Pierre Hornung, Cécile Lebrand

**Affiliations:** 1 Department of Fundamental Neurosciences, University of Lausanne, Rue du Bugnon 9, CH-1005 Lausanne, Switzerland; 2 Department of Ophthalmology, University of Lausanne, Hôpital ophtalmique Jules-Gonin, Av. de France 15, CH-1004 Lausanne, Switzerland; 3 Department of Psychiatry, Center for Psychiatric Neuroscience, Lausanne University Hospital, Prilly, CH-1008 Lausanne, Switzerland

## Abstract

The homeodomain transcription factor Nkx2.1 (NK2 homeobox 1) controls cell differentiation of telencephalic GABAergic interneurons and oligodendrocytes. Here we show that Nkx2.1 also regulates astrogliogenesis of the telencephalon from embryonic day (E) 14.5 to E16.5. Moreover we identify the different mechanisms by which Nkx2.1 controls the telencephalic astrogliogenesis. In Nkx2.1 knockout (*Nkx2.1*^−/−^) mice a drastic loss of astrocytes is observed that is not related to cell death. Further, *in vivo* analysis using BrdU incorporation reveals that Nkx2.1 affects the proliferation of the ventral neural stem cells that generate early astrocytes. Also, *in vitro* neurosphere assays showed reduced generation of astroglia upon loss of Nkx2.1, which could be due to decreased precursor proliferation and possibly defects in glial specification/differentiation. Chromatin immunoprecipitation analysis and *in vitro* co-transfection studies with an Nkx2.1-expressing plasmid indicate that Nkx2.1 binds to the promoter of glial fibrillary acidic protein (GFAP), primarily expressed in astrocytes, to regulate its expression. Hence, Nkx2.1 controls astroglial production spatiotemporally in embryos by regulating proliferation of the contributing Nkx2.1-positive precursors.

Proper forebrain development is carried out by a series of coordinated and regulated events involving controlled cell proliferation, differentiation, and guided migration of neuronal and glial cells. Several spatiotemporally orchestrated molecular mechanisms underlie the successful patterning of the telencephalon^1^,^2^,^3^,^4^,^5^,^6^,^7^. Both dorsal and ventral telencephalic regions are demarcated by specific gene expression patterns that then generate defined neuronal and glial populations. Dorsal progenitors express homeobox genes of the empty spiracles (*Emx1/Emx2*) and paired homeobox (*Pax6*) families, and the *atonal*-related genes Neurogenin (*Neurog) 1/Neurog2,* whereas the ventral progenitors are known to express homeobox genes of the *Nkx (Nkx2.1*) and distal-less (*Dlx1/Dlx2*) families, *Gsh1/2*, and *achaete-scute*-related gene *Ascl1*^8^,^9^,^10^,^11^,^12^.

Broadly, amongst the neuronal populations, the glutamatergic projection neurons are generated by dorsal telencephalic progenitors while the GABAergic (γ-aminobutyric acidergic) interneurons originate from the ventral telencephalic progenitors^3^,^13^,^14^,^15^. Among the glial populations, embryonic oligodendrocytes are produced in waves from the ventral telencephalic progenitors^16^,^17^. On the other hand, the exact timing of the generation and origin of the embryonic astroglial population is still a topic of active investigation. Since the astroglia play essential roles in guidance of forebrain commissures during embryonic brain development, a detailed understanding of the points of origin and exact timing of generation of the telencephalic astroglia is essential. In the ventral telencephalon, our and other reports have shown that early embryonic astrocytes are generated from ventral bipotential radial glia^18^,^19^. In the dorsal telencephalon, especially the cerebral cortex, astrocyte gliogenesis has been documented to occur only after neurogenesis (after E17 in mice), when the bipotential radial glial cells of the dorsal pallium differentiate into glia precursors or astrocytes^20^,^21^,^22^,^23^,^24^,^25^,^26^,^27^,^28^,^29^. Dorsal midline astroglia in the corpus callosum (CC), glial wedge (GW) and indusium griseum (IG) have also been shown to originate from the radial glia of the dorsomedial pallium^30^,^31^. However, the time of generation of some of the astrocytes occupying the CC and adjacent regions is noted to be between E13 and postnatal day 2 (P2), with a peak at E14, much earlier than previously proposed^30^. Later, the postnatal astrocytes that occupy the cerebral cortex region are believed to also originate from progenitor cells in the dorsolateral subventricular zone (SVZ)^32^,^33^. Recent evidence, however, shows that a population of locally differentiated glia in the postnatal cortex rather constitute the primary source of astrocytes^34^.

Nkx2.1, a homeodomain transcription factor, was initially found to regulate the transcription of many thyroid^35^,^36^,^37^ and lung-specific genes^38^,^39^. Interestingly, Nkx2.1 regulates several cell cycle related genes such as *Notch1, E2f3, Cyclin B1, Cyclin B2* and *c-Met* in developing embryonic lungs^40^. In the embryonic brain, Nkx2.1 controls the specification of the GABAergic interneurons and oligodendrocytes that populate the ventral and dorsal telencephalic region^3^,^16^,^37^,^41^,^42^,^43^. Loss of Nkx2.1 leads to ventral-to-dorsal re-specification of the pallium causing loss of GABAergic interneurons and oligodendrocytes in the dorsal telencephalic region^16^,^17^,^37^. Recently, we showed that Nkx2.1 regulates the generation of astrocytes that populate the ventral telencephalon during embryonic development and participates in axonal guidance in the anterior commissure^18^,^44^. We found that this Nkx2.1-derived astrocyte population is generated from three ventral telencephalic precursor regions, namely the medial ganglionic eminence (MGE), the anterior entopeduncular area (AEP)/preoptic area (POA) and the triangular septal nucleus (TS)^18^,^44^. Several works have revealed information on the origin of embryonic NG2 glia, also known as polydendrocytes or oligodendrocyte precursor cells^45^,^46^. Our work contributed by showing that embryonic NG2 glia originate from the Nkx2.1^+^ progenitors of the ventral telencephalon and promote precise blood vessel network development^44^. These cells have a highly complex branched morphology and are different from neurons, mature oligodendrocytes, astrocytes and microglia^45^,^46^,^47^,^48^,^49^,^50^,^51^,^52^. For a clear distinction, these cells will be referred to as “NG2 glia” in this study.

Here, we describe that Nkx2.1-derived astrocytes populate the corpus callosum (CC) and its surrounding regions in the embryonic dorsal telencephalon. These Nkx2.1-derived astrocytes are generated from E12.5 onwards with maximal production between E14.5 to E16.5. Interestingly, in *Nkx2.1*^−/−^ mice, we showed that the mutated Nkx2.1 (mut-Nkx2.1) leads to a drastic loss of astrocytes and NG2 glia in the dorsal telencephalic region at the midline. Since this mut-Nkx2.1 derived cell loss is not accompanied by increased cell death, we analyzed if cell proliferation of Nkx2.1^+^ progenitors in the three ventral precursor regions (MGE, AEP/POA and TS) was affected. *In vivo* BrdU incorporation showed that interestingly Nkx2.1 regulates astroglial generation by controlling the proliferation of Nkx2.1^+^ precursors. Similarly, *in vitro* neurosphere differentiation assays showed decreased generation of astroglia from Nkx2.1^−/−^ precursors, which may be a result of decreased proliferation of astroglial precursors upon loss of Nkx2.1 or perhaps defects in glial specification and/or differentiation. In addition, chromatin immunoprecipitation analysis showed that Nkx2.1 binds to the promoter of the glial fibrillary acidic protein (*GFAP*), primarily expressed in radial glia and astroglia. Co-transfection studies in HEK293 cells using tagged over-expressed Nkx2.1 and a mouse *GFAP* promoter construct confirmed that Nkx2.1 binds to the GFAP promoter and regulates expression of the *GFAP* gene. Thus, Nkx2.1 exhibits multilevel control over the generation of the dorsal telencephalic astroglia by spatially coordinating astroglial generation from three ventral precursor regions and temporally restricting maximal generation to E14.5 to E16.5. Further analysis into the complete repertoire of genes regulated by Nkx2.1 will shed light on the exact mode of action and help to delineate the different mechanisms involved in astrogliogenesis.

## Results

### Nkx2.1-derived astrocytes populate the dorsal telencephalon during development

**Figure 1:**
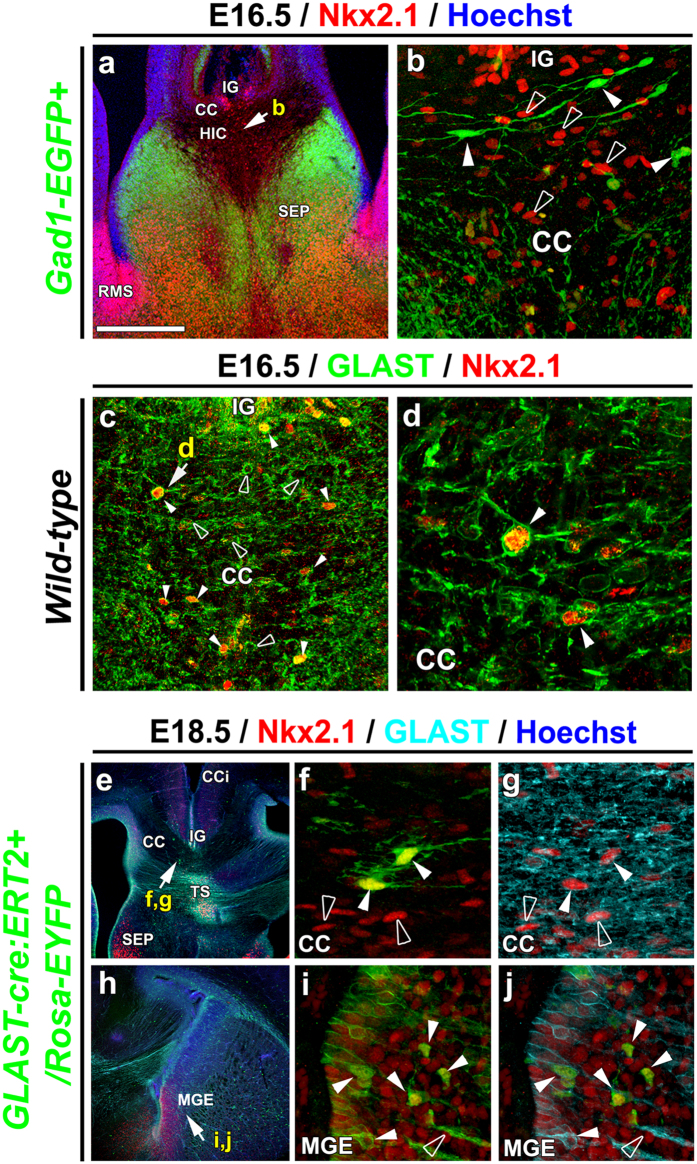
The Nkx2.1-positive cells of the CC are astroglial cells. (**a–d**) Double immunohistochemistry on coronal CC sections from *Gad1-EGFP*^+^ mice at E16.5 for GFP and Nkx2.1 (**a,b**) (n = 4), and on coronal CC sections from wild-type mice at E16.5 for GLAST and Nkx2.1 **(c,d)** (n = 2). (**e–j**) Triple immunohistochemistry for EYFP, Nkx2.1 and GLAST on coronal CC **(e-g)** and MGE (**h–j**) sections from *GLAST-Cre:ERT2*^+^*/Rosa-EYFP* mice at E18.5 (n = 5). Colocalization in the green and the red channels is yellow **(b,c,d,f,i** and **j)**. (**b)**, **(d)**, **(f)**, **(g), (i),** and **(j)** are higher magnifications of the CC and MGE region indicated by an arrow in (**a)**, **(c)**, **(e)** and **(h)**, respectively. Cell nuclei were counterstained in blue with Hoechst (**a,e**, and **h**). **(a–d)** At E16.5, several Nkx2.1^+^ (red) nuclei were observed in the medial part of the CC (open arrowheads in **b**). Nkx2.1 did not label the *Gad1-EGFP*^+^ interneurons (green) populating this region (solid arrowhead in **b)**. At this age, however, most of the Nkx2.1-expressing nuclei co-expressed astroglial markers like GLAST (solid arrowheads in **c** and **d)**. Quantification shows that 60% of E16.5 GLAST^+^ cells are Nkx2.1^+^ at E16.5 (n = 3) and at E18.5 (n = 3). **(e–j)** The Cre-mediated recombination was initiated by inducing the tamoxifen-inducible GLAST promoter at E14, and the GLAST-derived astroglia were visualized (in green) with the EYFP signal shown in **e,f** and **h,i,j**. Some GLAST^+^ astroglial cells (in light blue) in the CC **(e–g)** and the MGE **(i,j)** co-expressed Nkx2.1 (in red, solid arrowheads). Some of the GLAST^+^/Nkx2.1^+^ glia were not labeled by the EYFP signal and might have been generated before recombination was induced (open arrowheads in **f** and **g)**. **(CC)** corpus callosum; **(CCi)** cingulate cortex; **(IG)** induseum griseum; **(HIC)** hippocampal commissure; **(MGE)** medial ganglionic eminence; **(RMS)** rostral migratory stream; **(SEP)** septum; **(TS)** triangular septal nucleus. Bar = 675 μm in (**e)** and **(h)**; 450 μm in (**a)**; 67 μm in (**b)** and (**c)**; 40 μm in (**f)**, (**g)**, (**i)** and **(j)**; 30 μm in (**d**).

Nkx2.1-positive (Nkx2.1^+^) progenitors of the MGE, AEP/POA and septal nucleus generate embryonic GABAergic interneurons and oligodendrocytes that populate the ventral and dorsal telencephalon^3^,^16^,^37^,^41^,^42^,^43^. Our recent results have shown that Nkx2.1 additionally regulates the production of astrocytes and NG2 glia that populate the ventral telencephalon^18^,^44^. Further immunostaining of the subpallial transcription factor Nkx2.1 revealed strong expression in several differentiated cells within and around the CC in the dorsal telencephalon from E16.5 to E18.5 (n = 8; Fig. 1a–g). To differentiate the Nkx2.1^+^ cell types in the CC region we made use of several cell-type-specific transgenic strains.

To identify the GABAergic interneurons, we used *Gad1 (Glutamate decarboxylase 1*; also called as *GAD67)-EGFP* knock-in mice that express the enhanced green fluorescent protein (EGFP) in GAD67^+^ GABAergic interneurons^53^. In combination with Nkx2.1, as with previous observations, we show down-regulation of Nkx2.1 expression in dorsal telencephalic GABAergic interneuron population^54^ at E16.5, and also observe that none of the *Gad1-GFP*^+^ interneurons of the CC and dorsal surrounding areas express Nkx2.1 (n = 4; Fig. 1a,b; solid arrowheads in 1b).

To ascertain that Nkx2.1^+^ cells corresponded to NG2 glia, we made use of the *Cspg4 (Chondroitin sulfate proteoglycan 4)-Cre*^+^*/Rosa-EYFP* mice that express the enhanced yellow fluorescent protein (EYFP) in NG2^+^ (Neuron-glial antigen 2) glia^44^,^45^,^46^. We found that EYFP was expressed by Nkx2.1^+^ progenitors of the MGE (n = 3; Supp. Fig. S1a-c, solid arrowheads in b-c). However, the EYFP^+^ NG2 glia did not express the Nkx2.1 protein outside ventral germinal zones nor within the dorsal telencephalon (n = 3; Supp. Fig. S1g-i, open arrowheads in h-i). To further delineate the profile of Nkx2.1^+^ cells in the CC and the surrounding areas, we performed immunostaining against Nestin and GLutamate and ASpartate Transporter (GLAST), specific for post-mitotic astrocytes within the embryonic CC white matter^30^. Interestingly, from E16.5 to E18.5, 60% (n = 6) of the GLAST^+^ astrocytes cells of the CC and surrounding regions were found to be Nkx2.1^+^ (n = 11; Figs 1c,d,f,g and 3a,b,e, Supp. Fig. S1h-i, solid arrowheads). After postnatal day 0 (P0) however, Nkx2.1 was strongly down-regulated and not detected anymore in the astrocytes of the dorsal telencephalon (n = 8, not shown). Nkx2.1 expression was never detected in radial glial precursor cells of the glial wedge nor of the dorsal telencephalic ventricular zone detected by the aforementioned astrocytic markers (n = 11; Supp. Fig. S2 a-b).

Further analyses using tamoxifen-inducible *GLAST-Cre ERT*^*TM*^/*Rosa26-EYFP* mice displayed the presence of many EYFP^+^ early astrocytes outside of the germinal zones and also within the CC and surrounding area from E16.5 to E18.5 (n = 5; Fig. 1e–j). Many of these early astrocytes visualized by EYFP and GLAST co-staining showed Nkx2.1 expression also (n = 5; CC in Fig. 1f,g and MGE in 1i-j, solid arrowheads).

**Figure 2:**
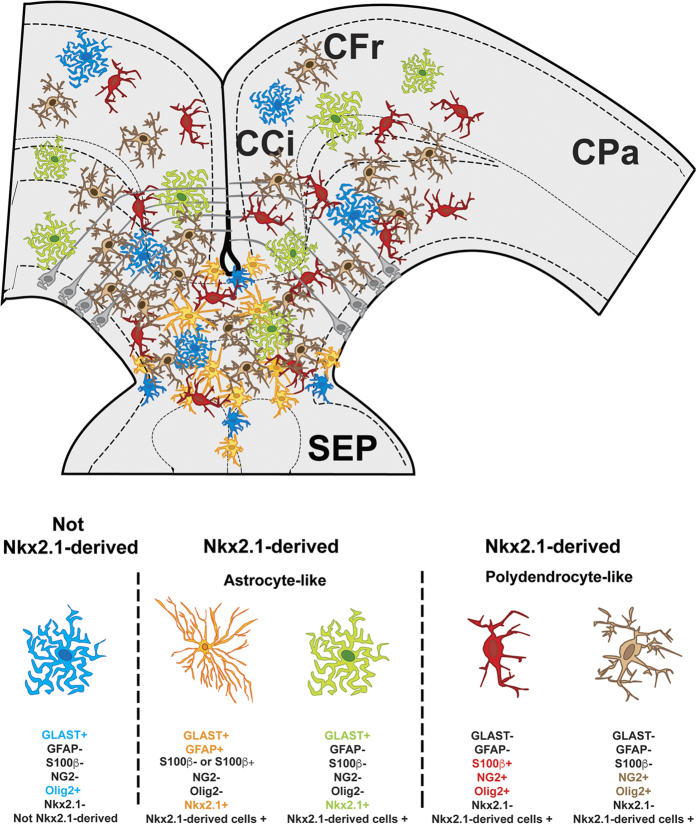
Four subtypes of *Nkx2.1*-derived glial cells. The schema represents a coronal view of the CC at E18.5 and summarizes the four different subtypes of *Nkx2.1*-derived glial populations visualized in our experiments. The CC forms a complex environment composed of one non-*Nkx2.1*-derived astroglial cell subtype and four different subtypes of *Nkx2.1*-derived glial cells. Three types of astrocyte-like cell populations are shown: in blue, the non-*Nkx2.1*-derived GLAST^+^/Olig2^+^/Nkx2.1^−^ cells, in orange, the *Nkx2.1*-derived GLAST^+^/GFAP^+^/S100β^+or−^/Olig2^−^/Nkx2.1^+^ cells and in green, the *Nkx2.1*-derived GLAST^+^/GFAP^−^/S100β^−^/Olig2^−^/Nkx2.1^+^ cells. Two types of *Nkx2.1*-derived polydendrocyte-like cells (also called NG2 glia) are shown; in red, the GLAST^−^/S100β^+^/NG2^+^/Olig2^+^/Nkx2.1^−^ cells, and in brown, the GLAST^−^/S100β^−^/NG2^+^/Olig2^+^/Nkx2.1^−^ cells. Under each glial cell-type picture, the expression profile of the different glial markers tested to characterize the glial cells, used in combination with the Nkx2.1 antibody, is presented. The (**+**) sign indicates that the glial cell type was labeled by the listed marker and the (–) sign indicates that the glial cell type was not labeled by the listed marker. **(CCi),** cingulate cortex; **(CFr)** frontal cortex; **(CPa)** parietal cortex; **(SEP)** septum.

**Figure 3:**
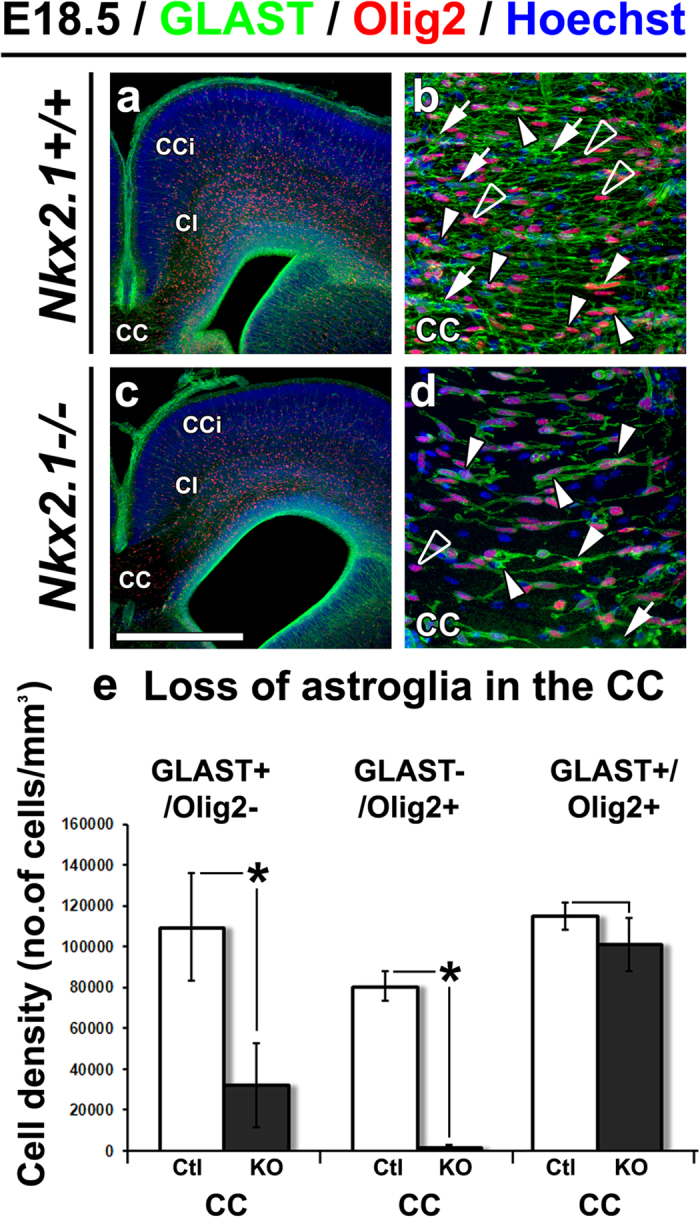
Loss of GLAST^+^/Olig2^−^ astroglia and GLAST^−^/Olig2^+^ NG2 glia in the CC of *Nkx2.1*^−/−^ mutant mice. **(a–d)** Double immunohistochemical staining with Olig2 and GLAST on CC coronal sections from: controls (Ctl), *Nkx2.1*^+/+^ and *Nkx2.1*^+/−^ (n = 4) **(a**,**b)**, and *Nkx2.1*^−/−^ (n = 2) **(c**,**d)** mice at E18.5. Cell nuclei were counterstained in blue with Hoechst **(a–d)**. (**b)** and **(d)** are higher magnifications of the CC of (**a)** and (**c)**, respectively. **(b** and **d)**. In the CC midline of *Nkx2.1*^−/−^ mice, there was a severe loss of GLAST^+^/Olig2^−^ astroglia (arrows) and GLAST^−^/Olig2^+^ NG2 glia (open arrowheads) but not of GLAST^+^/Olig2^+^ astroglia (solid arrowheads), compared to the control mice. **(e)** Bars (mean ± SEM from a sample of n = 3 control (Ctl) and n = 3 *Nkx2.1*^−/−^ mice) represent the density (number of cells/mm^3^) of GLAST^+^/Olig2^−^, GLAST^−^/Olig2^+^ and GLAST^+^/Olig2^+^ glial cells in the CC of *Nkx2.1*^−/−^ knockouts (KO) compared to Ctl mice at E18.5. The quantification confirms the significant decrease in GLAST^+^/Olig2^−^ astroglia (p-value = 0.0297) and GLAST^−^/Olig2^+^ NG2 glia (p-value = 0.0242) and no change in GLAST^+^/Olig2^+^ astroglia (p-value = 0.4469) in the *Nkx2.1*^−/−^ CC compared to control CC. **(CC)** corpus callosum; **(CCi)** cingulate cortex; **(CI)** cingulate bundle. Bar = 675 μm in (**a)** and (**c)**; 100 μm in (**b)** and (**d)**.

Therefore, the previous and current extensive immunohistochemical analyses in combination with different transgenic strains reveals that the CC and surrounding regions are populated with various Nkx2.1-derived glial cell types (summarized in Fig. 2)^18^. Presence or absence of Nkx2.1 protein expression primarily divides the Nkx2.1-derived glial classes into two major subtypes in both the dorsal and ventral telencephalon: astrocyte-like or polydendrocyte-like (or NG2 glia). The Nkx2.1-derived astrocyte-like population is further sub-divided into two populations: GLAST^+^/GFAP^+^/Nkx2.1^+^ (orange) and GLAST^+^/GFAP^-^/Nkx2.1^+^ (green) (Fig. 2 and Supp. Fig. S3). The polydendrocyte-like population (or NG2 glia) is also further sub-divided into two populations: NG2^+^/Olig2^+^/S100β^+^/Nkx2.1^−^ (red) and NG2^+^/Olig2^+^/S100β^−^/Nkx2.1^−^ (brown) populations (Fig. 2 and Supp. Fig. S3). Interestingly, a subpopulation of the GLAST^+^ astrocyte-like population within the telencephalon (blue) is not Nkx2.1-derived and expresses Olig2 (n = 4; Fig. 2; Fig. 3a,b and e)^18^.

**Figure 4:**
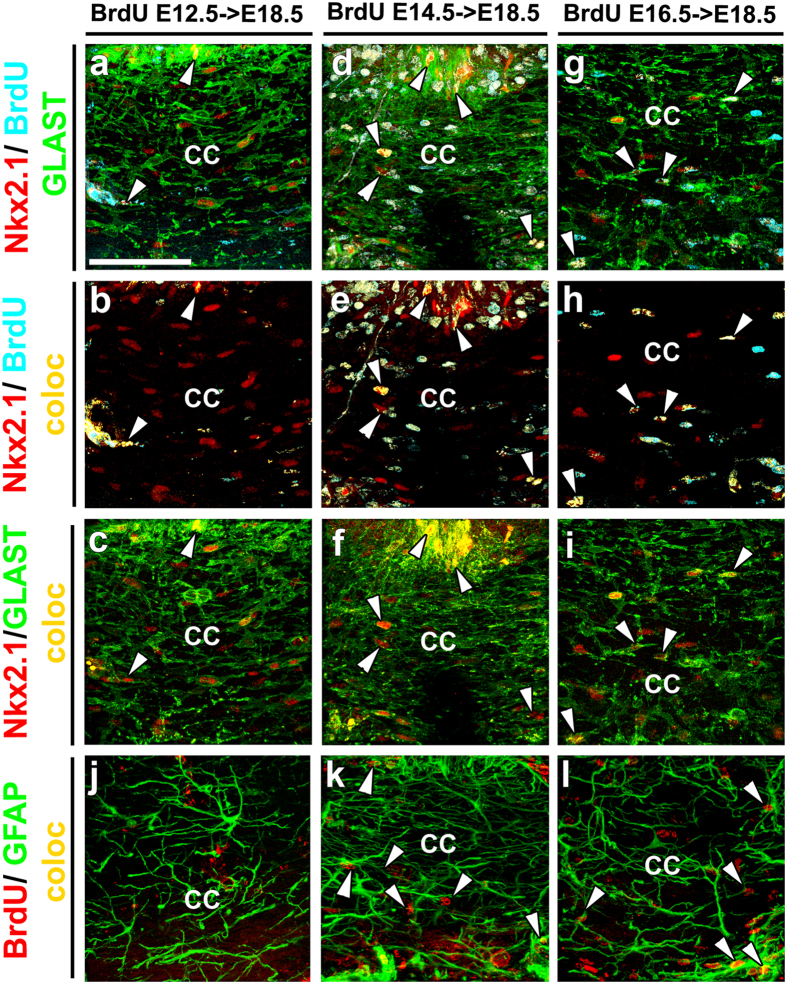
Nkx2.1-positive astroglia of the CC are generated between E14.5 and E16.5. **(a–i)** Triple immunohistochemistry with Nkx2.1, 5-bromo-2′-deoxyuridine (BrdU), and GLAST, and **(j–l)** double immunohistochemistry with BrdU and GFAP on corpus callosum (CC) coronal sections from wild type brains injected at E12.5 (n = 2) **(a–c** and **j)**, E14.5 (n = 2) **(d–f** and **k)** and E16.5 (n = 2) **(g–i** and **l). (a–i)** At E18.5, several GLAST^+^ astroglial cells (green) expressing Nkx2.1 (red) are present in the CC midline. **(b**,**e** and **h)** Colocalization between the blue (BrdU) and the red (Nkx2.1) channel is observed in yellow. **(c**,**f** and **i)** Colocalization between the green (GLAST) and the red (Nkx2.1) channel is observed in yellow. The solid arrowheads show the Nkx2.1^+^/GLAST^+^/BrdU^+^ cells revealing that the bulk of division for the Nkx2.1^+^ astroglial cells of the CC occurs between E14.5 **(e)** and E16.5 **(h)**. Quantification indicates that the fraction of Nkx2.1^+^/GLAST^+^ glia that show BrdU labeling at E18.5 is 4% when BrdU injections are performed at E12.5, 33% when BrdU injections are performed at E14.5, and 45% when BrdU injections are performed at E16.5. **(j–l)** Numerous GFAP^+^ astroglial cells (in green) are present in the CC midline. Colocalization between the green (GFAP) and the red (BrdU) channel is observed in yellow. The solid arrowheads signify GFAP^+^/BrdU^+^ cells indicating that main division of GFAP^+^ glial cells in the CC occurs also from E14.5 **(k)** to E16.5 (**l)**. Quantification indicates that the fraction of GFAP^+^ glia that show BrdU labeling at E18.5 is 33% when BrdU injections are performed at E12.5, 60% when BrdU injections are performed at E14.5, and 70% when BrdU injections are performed at E16.5. Bar = 60 μm in (**a–l**).

Nkx2.1-derived astrocyte-like cells populate the CC region towards the end of the embryonic period. Use of tamoxifen-inducible *GLAST-Cre ERT*^*TM*^/*Rosa26-EYFP* mice showed several Nkx2.1^+^/GLAST^+^/EYFP^+^ astrocytes beginning from E14.5 onwards as tamoxifen was delivered at E14.5 (n = 5; Fig. 1e–g). To decipher the exact timing of generation of these Nkx2.1^+^ glial cells in the CC region, we administered 5-bromo-2′-deoxyuridine (BrdU) to wild-type (WT) pregnant females at E12.5 (n = 2), E14.5 (n = 2), and E16.5 (n = 2), and analyzed BrdU incorporation at E18.5 in CC astrocytes co-expressing Nkx2.1 and GLAST or GFAP (Fig. 4). Combined immunostaining revealed a few BrdU^+^/Nkx2.1^+^/GLAST^+^and BrdU^+^/GFAP^+^ embryonic astrocytes in the CC when BrdU was delivered at E12.5 (Fig. 4a–c and j, solid arrowheads). The bulk of the BrdU^+^/Nkx2.1^+^/GLAST^+^ (33% at E14.5 and 45% at E16.5) and BrdU^+^/GFAP^+^ (60% at E14.5 and 70% at E16.5) embryonic astrocytes were observed when BrdU was delivered at E14.5 (Fig. 4d–f and k, solid arrowheads) and E16.5 (Fig. 4h,i and l, solid arrowheads) indicating that the majority of Nkx2.1-derived astrocytes in the CC region are produced between E14.5 and E16.5.

The results indicate that Nkx2.1 expression is primarily maintained in the astrocyte population of the CC region from E14.5 to E18.5.

### Nkx2.1 controls gliogenesis in the embryonic telencephalon

**Figure 5:**
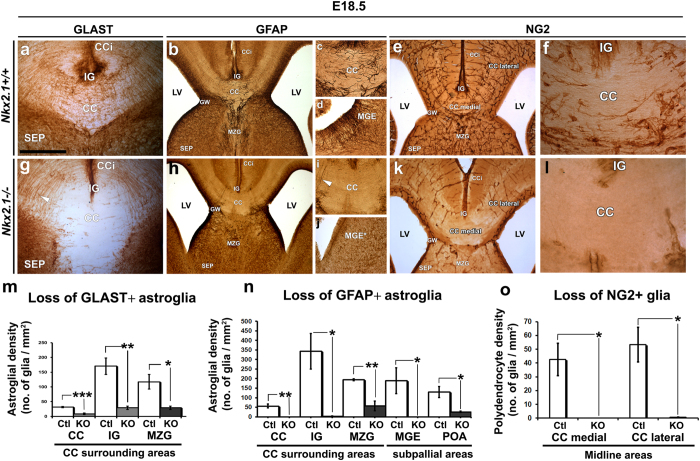
Loss of different glial cell types in the CC, medial cortical areas and subpallium of *Nkx2.1*^−/−^ mice brains. DAB staining with GLAST (n = 4 for *Nkx2.1*^+/+^ or *Nkx2.1*^+*/*−^ controls (Ctl) and 3*Nkx2.1*^−/−^) **(a** and **g)**, GFAP (n = 4 for Ctl and 3 for *Nkx2.1*^−/−^) **(b–d** and **h–j)** and NG2 (n = 5 for Ctl and n = 4 for *Nkx2.1*^−/−^) **(e,f** and **k,l)** on CC and MGE coronal sections from controls (Ctl) *Nkx2.1*^+/+^ or *Nkx2.1*^+/−^ **(a**–**f)** and *Nkx2.1*^−/−^ **(g**–**l)** mice at E18.5. (**c), (f), (i)** and (**l)** are higher magnifications of the CC region seen in (**b**), (**e**), (**h**) and (**k)**, respectively. **(d)** and (**j)** are higher magnifications of the MGE region seen in (**b)** and (**h)**, respectively. DAB staining for GLAST, GFAP and NG2 revealed a drastic loss of astroglial and polydendroglial cell types from the CC and surrounding areas and from the MGE of the *Nkx2.1*^−/−^ mice compared to control mice (compare **g** with **a**;**h,i** with **b,c**;**j** with **d** and **k,l** with **e,f**). Only the GFAP^+^ radial glial cells originating from the Nkx2.1^−^ glial wedge (GW) bordering the CC remain unaffected (white arrowhead in **g** and **i**). **(m,n** and **o)** Bars (mean ± SEM from a sample of n = 4 brains in Ctl and n = 3 brains in *Nkx2.1*^−/−^ for GLAST and GFAP and of n = 5 in Ctl and n = 3 in *Nkx2.1*^−/−^ for NG2) represent the cell densities of GFAP^+^ or NG2^+^ glial cells/mm^2^. The quantification of the GLAST^+^ (p-value = 0.0005 for CC, 0.0036 for IG, 0.028 for MZG), GFAP^+^ (p-value = 0.0056 for CC, 0.0216 for IG, 0.0067 for MZG, 0.0496 for MGE, and 0.0247 for POA) and NG2^+^ (p-value = 0.0334 for CC medial and 0.0191 for CC lateral) glial cell density showed a drastic and significant loss of these cells in the CC and surrounding areas as well as in medial cortical areas of the *Nkx2.1*^−/−^ brains compared to the Ctl brains. **(CC)** corpus callosum; **(CCi)** cingulate cortex; **(GW)** glial wedge; **(IG)** induseum griseum; **(LV)** lateral ventricle; **(MZG)** midline zipper glia; **(MGE)** medial ganglionic eminence; **(SEP)** septum. Bar = 500 μm in (**b)**, (**e)**, (**h)** and (**k)**; 250 μm in (**a)**, (**c)**, (**d), (f),** (**i), (j)** and (**l)**; 125 μm in (**f)** and (**l).**

To further investigate the function of Nkx2.1 in regulating embryonic gliogenesis, we performed immunohistochemistry at E18.5 for astroglia and NG2 glia markers in control and *Nkx2.1*^−/−^ embryos expressing an inactivated truncated Nkx2.1 (mut-Nkx2.1) (Fig. 5). As controls we used both homozygous (*Nkx2.1*^+/+^) and heterozygous (*Nkx2.1*^+/−^) mice. In control mice, GLAST^+^ (n = 2) or GFAP^+^ (n = 4) astroglia, and NG2^+^ glia (n = 5) were clearly visible in the CC and surrounding regions, in the medial cortical area as well as in the septum (SEP) (Fig. 5a–c and e,f, respectively). In contrast we observed a drastic loss (70 to 100%) of astroglia (n = 2 stained for GLAST; n = 3 stained for GFAP) and NG2 glia (n = 4) in all midline dorsal regions in *Nkx2.1*^−/−^ mice (Fig. 5g–i and k,l). However, Nkx2.1 inactivation did not affect the number and organization of radial glia within the GW as expected for glial cells that do not express Nkx2.1 (compare Fig. 5a,b and g,h). Quantitative measurements with the astrocyte marker, GLAST (Fig. 5m), GFAP (Fig. 5n) and NG2 (Fig. 5o) confirmed the severe loss of astroglia and NG2 glia in the midline dorsal telencephalic areas in *Nkx2.1*^−/−^ mice. There was a particularly high loss of GLAST^+^ and GFAP^+^ astrocytes in the CC and surrounding areas (indusium griseum, IG and midline zipper glia, MZG) in the *Nkx2.1*^−/−^ embryos (n = 3) compared to control embryos (n = 4) (p-value < 0.05 for CC, IG and MZG, Fig. 5m and n). Interestingly, GFAP labeling also revealed the loss of a subpopulation of GFAP^+^ radial glial precursors within the ventral telencephalon, in the mutant-MGE (MGE*; mutant precursor regions are denoted by an additional asterisk^37^) and mutant-POA (POA*) ventricular zone (VZ) of the *Nkx2.1*^−/−^ embryos (compare Fig. 5d to j, p-value = 0.0496 for MGE and 0.0247 for POA; Fig. 5n). Additionally there was a near complete loss (99 to 100%) of NG2 glia in the medial cortical areas of *Nkx2.1*^−/−^ embryos (n = 3) compared to control embryos (n = 5) (p-value = 0.0334 for CC medial and 0.0191 for CC lateral; Fig. 5k,l and o).

As we identified different *Nkx2.1*-derived glial cell populations, Olig2^+^ or Olig2^−^ astrocytes and Olig2^+^ NG2 glia, we further investigated the function of Nkx2.1 in astrogliogenesis in the different glial cell types (Fig. 2). For this, we performed immunostaining with Olig2 and GLAST on telencephalic CC sections from both controls (n = 4) and *Nkx2.1*^−/−^ (n = 2) mice. In the *Nkx2.1*^−/−^ CC, a significant reduction (around 70%) of GLAST^+^/Olig2^−^ astrocytes was observed compared to controls (p-value = 0.0297) while no difference was detected for GLAST^+^/Olig2^+^ astroglial cell density (p-value = 0.4469) (Fig. 3a–e). The GLAST^−^/Olig2^+^ NG2 glia population was almost completely lost (around 98%) in the CC (p-value = 0.0242; Fig. 3e). In summary, Nkx2.1 regulates both the GLAST^+^/Olig2^–^ astroglia and GLAST^−^/Olig2^+^ NG2 glia but not the GLAST^+^/Olig2^+^ astroglia (also illustrated in Fig. 2).

To investigate if cell death is the reason for the marked decrease in the number of glial cells observed, we analyzed the control (n = 4 for CC region; n = 5 for POA region) and *Nkx2.1*^−/−^ (n = 6 for CC region; n = 10 for POA region) brains at E16.5 for presence of cleaved-caspase 3, a key biomarker of apoptosis (Supp. Fig. S4a–d and S4i) as well as terminal deoxynucleotidyl transferase-mediated dUTP-biotin nick end labeling (TUNEL) assay to detect DNA fragmentation that results from different cell death processes (n = 16 for CC in controls, n = 22 for CC in knockouts; n = 6 for POA in controls, n = 5 for POA in knockouts; n = 10 for MGE in controls, n = 11 for MGE in knockouts; n = 7 for SEP in controls, n = 14 for SEP in knockouts; Supp. Fig. S4e-h and S4j). Hoechst was also used to visualize pyknotic nuclei. Quantification of dying cells labeled by cleaved-caspase 3 (Supp. Fig. S4i), and by TUNEL staining (Supp. Fig. S4j), revealed no significant differences between the *Nkx2.1*^−/−^ brains and the control brains in any of the telencephalic regions tested namely the CC, MGE, POA or septum (p-value = 0.1225 for CC and 0.4618 for POA with cleaved caspase 3 staining; p-value = 0.7934 for CC, 0.8193 for POA, 0.4032 for MGE, and 0.4879 for SEP with TUNEL assay). The size and morphology of cell nuclei were comparable in both control and mutant brains.

In conclusion, glial cells occupying the CC are under the regulation of Nkx2.1. Moreover, the significant loss of astrocytes and NG2 glia in the *Nkx2.1*^−/−^ telencephalon is not due to glial cell death.

### Nkx2.1 regulates the proliferation of astrocyte ventral progenitors in embryonic brains

The loss of specified glia in *Nkx2.1*^−/−^ mice may be due to insufficient proliferation of ventral glial Nkx2.1-precursors in the progenitor zones, MGE, AEP/POA and TS. We labeled coronal sections of the ventral precursor regions in both *Nkx2.1*^+/+^ or *Nkx2.1*^+/−^ controls (n = 4) and *Nkx2.1*^−/−^ (n = 4) mice brains at E16.5 with Nkx.2.1 and radial glia/astrocytic marker, GLAST. Both, the mutated Nkx2.1 (mut-Nkx2.1) and the WT Nkx2.1 proteins are similarly recognized by anti-Nkx2.1 antibody indicating that the mutated protein conserves an intact epitope sufficient for recognition by the antibody^55^.

**Figure 6:**
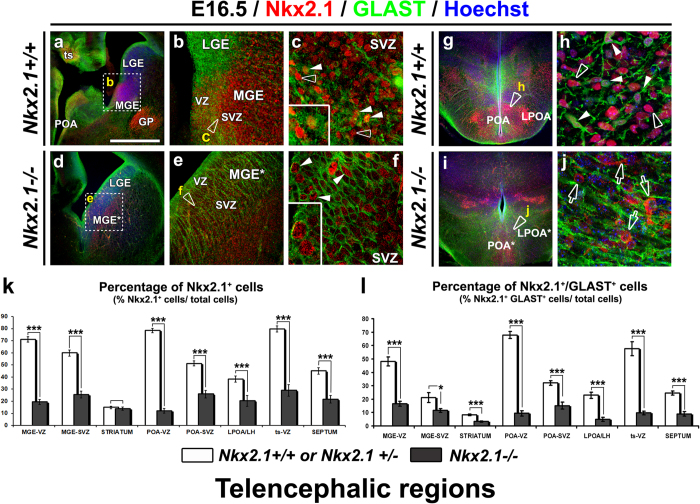
Incapacity of precursor cells to generate astrocytes in the *Nkx2.1*^−/−^ mouse brain. **(a–c** and **g,h)** Double immunohistochemical staining with Nkx2.1 and GLAST on MGE **(a–c)** and POA **(g,h)** coronal sections from *Nkx2.1*^+/+^ or *Nkx2.1*^+/−^ control (Ctl) (n = 4) brains at E16.5 **(d–f** and **i,j)** Double immunohistochemical staining for mutated-Nkx2.1 (mut-Nkx2.1) and GLAST on MGE* **(d–f)** and POA* **(I,j)** coronal sections from *Nkx2.1*^−/−^ (n = 4) brains at E16.5. Cell nuclei were counterstained in blue with Hoechst **(a,d,g,h and i,j)**. (**b), (c), (e), (f), (h)** and **(j)** are higher magnifications of the regions seen in (**a)**, **(d)**, (**g)** and (**i)** respectively. **(a–c)** In the germinal regions of the Ctl MGE, numerous Nkx2.1^+^ progenitors were GLAST^+^ (solid arrowheads and inset in **c**), while some were GLAST^−^(open arrowheads in **c**). **(d–f)** In *Nkx2.1*^−/−^ MGE* germinal regions, only few GLAST^+^ progenitors expressed the mut-Nkx2.1 protein (solid arrowheads and inset in **f**). **(g–h)** In the parenchyma of Ctl POA, many GLAST^+^ astroglial cells (solid arrowheads in **h**) and neurons expressed Nkx2.1. **(i,j)** In the parenchyma of *Nkx2.1*^−/−^ POA*, GLAST^+^ astroglial cells expressing the mut-Nkx2.1 protein disappeared and only a few neurons expressing mut-Nkx2.1 protein were observed (open arrows in **j**). **(GP)** globus pallidus; **(LGE)** lateral ganglionic eminence; **(LPOA)** lateral POA; **(MGE)** medial ganglionic eminence; (MGE*) mutant MGE; **(POA)** preoptic area; (POA*) mutant POA; **(SEP)** septum; **(SVZ)** subventricular zone; **(TS)** triangular septal nucleus, **(VZ)** ventricular zone. Bar: 100 μm in (**b)** and (**e)**; 45 μm in (**c)** and (**f)** and 50 μm in (**h)** and (**j)**. **(k,l)** Bars (mean ± SEM from n = 4 Ctl and n = 4 *Nkx2.1*^−/−^ brains) represent the percentage of Nkx2.1^+^ cells **(k)** and Nkx2.1^+^/GLAST^+^ precursors and astroglial cells **(l)** in Ctl (white-columns) and *Nkx2.1*^−/−^ (black-columns) regions at E16.5. **(k** and **l)** The number of cells **(k)** and GLAST^+^ precursors and post-mitotic cells **(l)** expressing mut-Nkx2.1 were drastically decreased in all the subpallial telencephalic regions of the *Nkx2.1*^−/−^ brains. **(k)** p-values < 0.0001 for MGE-VZ/-SVZ, POA-VZ/-SVZ, ts-VZ, SEPTUM, < 0.0005 for LPOA/LH, and 0.571 for STRIATUM. **(l)** p-values < 0.0001 for MGE-VZ, STRIATUM, POA-VZ/-SVZ, LPOA/LH, ts-VZ, SEPTUM and 0.0139 for MGE-SVZ. (***p < 0.001; **p < 0.01; *p < 0.05; Student’s *t-*test).

In the VZ, SVZ and mantle zone of the control MGE, many GLAST^+^ precursors and differentiated astroglia co-express Nkx2.1 (Fig. 6a–c and h, solid arrowheads) whereas in the germinal and mantle zones of the mutant MGE* in *Nkx2.1*^−/−^ mice very few GLAST^+^ precursors and astroglia showed co-localization with the mut-Nkx2.1 (Fig. 6d–f, solid arrowheads). This difference may be attributed as shown previously, to the fact that although a MGE-like structure forms in the mutant (MGE*), it has been re-specified to a more dorsal LGE (lateral ganglionic eminence)-like fate^38^. A similar reduction in cells containing GLAST and mut-Nkx2.1 were seen in the mutant POA* of *Nkx2.1*^−/−^ mice (Fig. 6i,j). Quantitative analysis revealed a very large and significant decrease of the total number of precursors (50 to 85%) and specifically GLAST^+^ precursors (45 to 86%) expressing mut-Nkx2.1 in the VZ, SVZ of the MGE*, POA* and TS* (p-value < 0.0001 in the VZ of MGE, POA and TS in Fig. 6k,l and p-value = 0.0139 for the SVZ of MGE in Fig. 6k). Consequently, the number of GLAST^+^ differentiated astrocytes co-expressing mut-Nkx2.1 in the parenchyma (striatum (ST); lateral preoptic area (LPOA)/Lateral hypothalamus (LH); septum (SEP)) in *Nkx2.1*^−/−^ mice (n = 4) was severely decreased (60 to 80%) compared to control mice (n = 4) (p-value < 0.0001 for striatum, LPOA and septum in Fig. 6l). Therefore, mutation of Nkx2.1 results in the severe loss of precursors and differentiated astrocytes in the brains.

**Figure 7:**
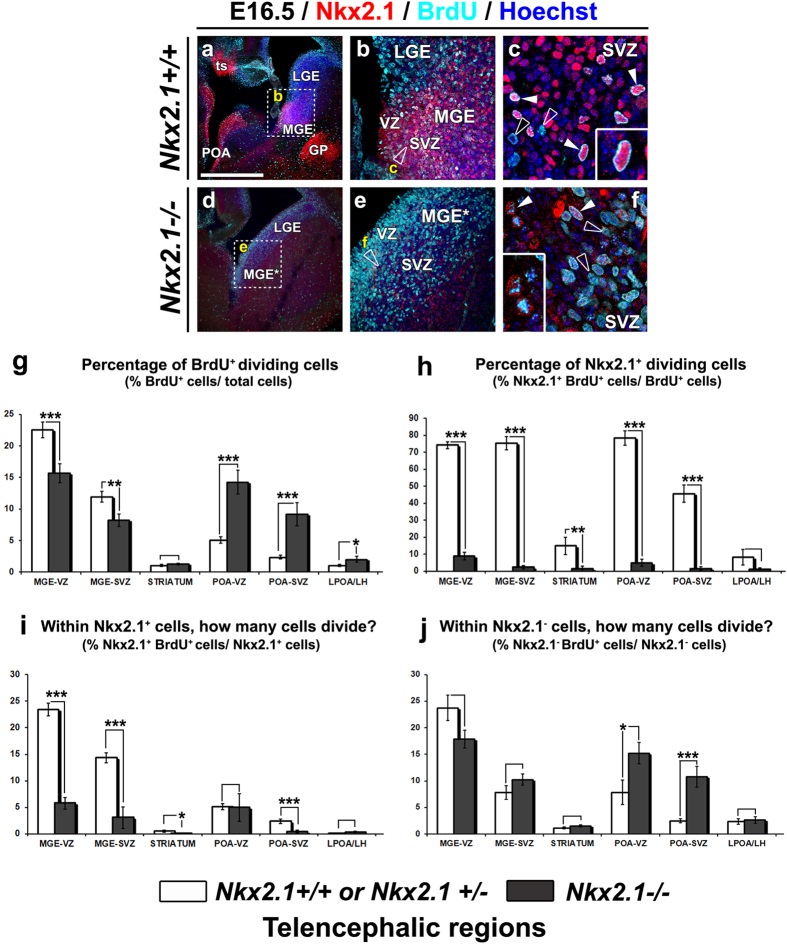
Incapacity of Nkx2.1^+^ precursor cells to divide after Nkx2.1 inactivation. Double immunohistochemical staining with Nkx2.1 and BrdU **(a–c)** and mut-Nkx2.1 and BrdU **(d–f)** on telencephalic coronal sections from *Nkx2.1*^+/+^ or *Nkx2.1*^+/−^ control (Ctl) (n = 4) **(a–c)** and *Nkx2.1*^−/−^ (n = 4) **(d–f)** mice brains at E16.5. Cell nuclei were counterstained in blue with Hoechst **(a–f)**. (**b)** and (**e)** are higher-magnifications of MGE squared regions in (**a)** and (**d)**, respectively. (**c)** and (**f)** are higher-magnifications of MGE in **b** and **e**, respectively. **(a–c)** In VZ and SVZ of Ctl MGE, AEP/POA and TS, numerous Nkx2.1^+^ precursors (red) were BrdU^+^ (light blue) (solid arrowheads and inset in **c**). Other BrdU^+^ dividing cells were Nkx2.1^−^ (open arrowheads, **c**). **(d–f)** In the VZ and SVZ of the *Nkx2.1*^−/−^ MGE*, numerous precursors were BrdU^+^ (open arrowheads, **f**), but only few were mut-Nkx2.1^+^ (solid arrowheads and inset, **f**). **(LGE),** lateral ganglionic eminence; **(MGE),** medial ganglionic eminence; (MGE*) mutant MGE; **(POA)** preoptic area; **(SVZ)** subventricular zone; **(TS)** triangular septal nucleus; **(VZ)** ventricular zone. Bar = 675 μm in (**a)** and **(d)**; 100 μm in (**b)** and (**c)**; 45 μm in (**c)** and (**d)**. **(g–j)** Bars (mean ± SEM from n = 4 Ctl and *Nkx2.1*^−/−^ brains each) represent the percentage of the BrdU^+^ cells **(g)**; the percentage of Nkx2.1^+^ or mut-Nkx2.1^+^ dividing cells amongst the total BrdU^+^ cells **(h)**; the percentage of BrdU^+^ cells amongst the total Nkx2.1^+^ or mut-Nkx2.1^+^ cells **(i),** and the percentage of Nkx2.1^−^ cells co-stained for BrdU **(j)** in Ctl and *Nkx2.1*^−/−^ regions at E16.5. **(g)** A significant decrease of BrdU^+^ precursors in MGE* was balanced by a significant increase in BrdU^+^ precursors in POA* of *Nkx2.1*^−/−^ mice; **(h)** a drastic and significant decrease in the mut-Nkx2.1^+^ dividing cells in all *Nkx2.1*^−/−^ regions; **(i)** the mut-Nkx2.1^+^ cells lost their capacity to divide in the MGE*, striatum and SVZ-POA*; **(j)** mut-Nkx2.1^−^ cells still divided normally in the MGE* and POA* of *Nkx2.1*^−/−^ mice. **(g–j)** Corresponding p-values for all the quantifications are indicate within text describing the results (***p < 0.001; **p < 0.01; *p < 0.05; Student’s *t-*test).

To test the cell proliferation status of precursors in the germinal zones we used a marker of S-phase, BrdU. The rate of cell proliferation was examined in *Nkx2.1*^+/+^ (n = 8) or *Nkx2.1*^+/−^ (n = 3) controls and *Nkx2.1*^−/−^ (n = 8) mice at E16.5 when the bulk of the embryonic telencephalic glia is generated. In the control brains, numerous Nkx2.1^+^ precursors were co-labeled with BrdU in both the VZ and SVZ of the MGE, (Fig. 7a–c, solid arrowheads). For quantification, we used n = 4 controls and n = 4 knockouts (Fig. 7g–j). In the mutant MGE* of *Nkx2.1*^−/−^ mice, we quantified significantly less BrdU^+^ progenitors in the VZ and SVZ compared to the control MGE (p-values < 0.001 for MGE-VZ; 0.0064 for MGE-SVZ, 0.235 for STRIATUM; and 0.0126 for LPOA/LH in Fig. 7g). Intriguingly, an increased number of total BrdU^+^ cells were found in the POA* VZ and SVZ regions in *Nkx2.1*^−/−^ mice (p-values < 0.001 for POA-VZ/-SVZ). Nonetheless, BrdU^+^ precursors only very rarely expressed mut-Nkx2.1 in both the mutant MGE* and POA* regions (compare Fig. 7c to f). The number of BrdU^+^ dividing cells labeled with mut-Nkx2.1 was severely reduced (75 to 80%) in the *Nkx2.1*^−/−^ brains (p-values < 0.0001 for MGE-VZ/-SVZ and POA-VZ/-SVZ; 0.0042 for STRIATUM; and 0.3182 for LPOA/LH in Fig. 7h). We also quantified the percentage of Nkx2.1^+^ and mut-Nkx2.1^+^ cells co-stained for BrdU. A clear reduction in the percentage of mut-Nkx2.1^+^/BrdU^+^ proliferating cells was seen in *Nkx2.1*^−/−^ mice (Fig. 7i). Hence, mutation of Nkx2.1 results in the incapacity of the Nkx2.1-derived precursors to divide (p-values < 0.0001 for MGE-VZ/-SVZ;<0.0005 for POA-SVZ; 0.043 for STRIATUM; 0.934 for POA-VZ; and 0.3518 for LPOA/LH in Fig. 7i). In contrast, dividing cells that did not express mut-Nkx2.1 in the *Nkx2.1*^−/−^ brains were either not affected in the mutant MGE* or up regulated in the mutant POA* (p-values < 0.0001 for POA-SVZ; 0.0214 for POA-VZ; 0.0796 for MGE-VZ, 0.1503 for MGE-SVZ; 0.1015 for STRIATUM; and 0.741 for LPOA/LH in Fig. 7j).

Altogether, these observations indicate that the transcription factor Nkx2.1 controls the proliferation step of Nkx2.1^+^ precursors in the MGE and the POA, the subpallial domains that mainly generate early embryonic astroglia.

### Nkx2.1 regulates the production of Nkx2.1-derived astroglia in embryonic brains

**Figure 8:**
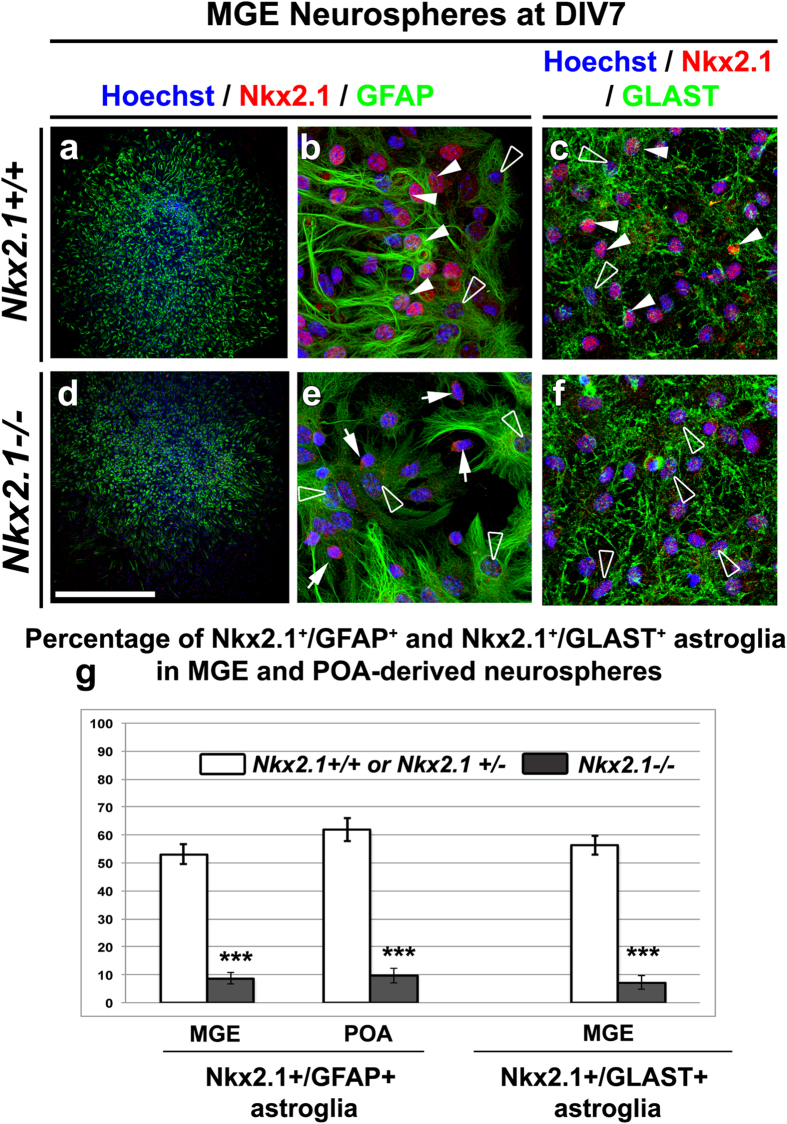
Nkx2.1^+^ MGE and POA stem cells do not differentiate into astrocytes after *Nkx2.1* inactivation. Double immunocytochemistry with: Nkx2.1 and GFAP **(a** and **b)** or with: Nkx2.1 and GLAST (**c**) on MGE-derived neurospheres from E14.5 *Nkx2.1*^+/+^ or *Nkx2.1*^+*/*−^ control (Ctl) mice (n = 8) after 7 days *in vitro* (DIV). Double immunocytochemistry for mut-Nkx2.1 and GFAP **(d** and **e)** or for mut-Nkx2.1 and GLAST on MGE*-derived neurospheres from E14.5 *Nkx2.1*^−/−^ mice (n = 6) after 7 days DIV. Cell nuclei were counterstained in blue with Hoechst **(a–f)**. (**b)** and **(e)** are higher magnifications of the regions seen in **a** and **d** respectively. In neurospheres derived from control MGE, numerous GFAP^+^ and GLAST^+^ astrocytes were labeled with Nkx2.1 (solid arrowheads in **b** and **c**), while others were not (open arrowheads in **b** and **c**). In contrast in neurospheres derived from *Nkx2.1*^−/−^ MGE*, GFAP^+^ and GLAST^+^ astrocytes were never observed to be co-labeled with mut-Nkx2.1 (open arrowheads in **e** and **f**), but neuronal cells still express the mutated Nkx2.1 protein (arrows in **e**). Bar = 675 μm in **a** and **d,** and 50 μm in **b,c,e** and **f**. **(g)** Bars represent the percentage of GFAP^+^ or GLAST^+^ astrocytes labeled with Nkx2.1 in MGE– or POA–derived neurospheres from Ctl and of GFAP^+^ or GLAST^+^ astrocytes labeled with mutated Nkx2.1 in MGE*–derived neurospheres from *Nkx2.1*^−/−^ mice brains (mean ± SEM from a sample of n = 21 MGE, n = 34 POA Ctl neurospheres and n = 22 MGE*, n = 28 POA* *Nkx2.1*^−/−^ neurospheres for GFAP staining; mean ± SEM from a sample of n = 5 MGE Ctl neurospheres and n = 3 MGE* *Nkx2.1*^−/−^ neurospheres for GLAST staining). Neurospheres originating from *Nkx2.1*^−/−^ MGE* and POA* practically lost the capacity to produce Nkx2.1-derived astrocytes (p-value < 0.0001 for both). (***p < 0.001, Student’s *t-*test).

Next, we analyzed the *in vitro* capacity of Nkx2.1^+^ precursors to generate early astroglial cells by observing the MGE- and POA-derived neurosphere differentiation at E14.5. After 7 days *in vitro* (DIV), control MGE and POA neurospheres are able to differentiate and generate several brain cell types^56^ which are GFAP^+^ (Fig. 8b and e) and GLAST^+^ (Fig. 8c and f) astrocytes as well as ßIII-tubulin^+^ post-mitotic neurons (not shown). GFAP^+^ and GLAST^+^ astrocytes were uniformly dispersed over the entire surface of the spheres (Fig. 8a and d). Labeling with Nkx2.1 showed that in the neurospheres derived from control MGE and POA, Nkx2.1 was expressed in the nucleus of about 50% of the GFAP^+^ and GLAST^+^ astroglia (Fig. 8b and g; solid arrowheads). However, *in vitro* differentiation of E14.5 *Nkx2.1*^−/−^ mutant MGE* and POA*-derived neurospheres revealed that precursors expressing mut-Nkx2.1 nearly lost the capacity to generate mut-Nkx2.1^+^/GFAP^+^ and mut-Nkx2.1^+^/GLAST^+^ astroglia (Fig. 8e–f). Quantification showed that mutation of Nkx2.1 in the MGE* and POA* neurospheres of *Nkx2.1*^−/−^ induced a significant decrease of mut-Nkx2.1^+^/GFAP^+^ and mut-Nkx2.1^+^/GLAST^+^ astroglia (p-value < 0.0001 in the MGE* and POA* neurospheres in Fig. 8g). While more than 50% of differentiated GFAP^+^ and GLAST^+^ astroglia expressed Nkx2.1 in control neurospheres, less than 10% of GFAP^+^ and GLAST^+^ astroglia expressed mut-Nkx2.1 in mutant neurospheres (Fig. 8g) indicating that in mutant MGE* and POA* neurospheres mut-Nkx2.1^+^ precursors have mainly lost their capacity to generate astroglia.

### Nkx2.1 directly regulates the expression of GFAP

**Figure 9:**
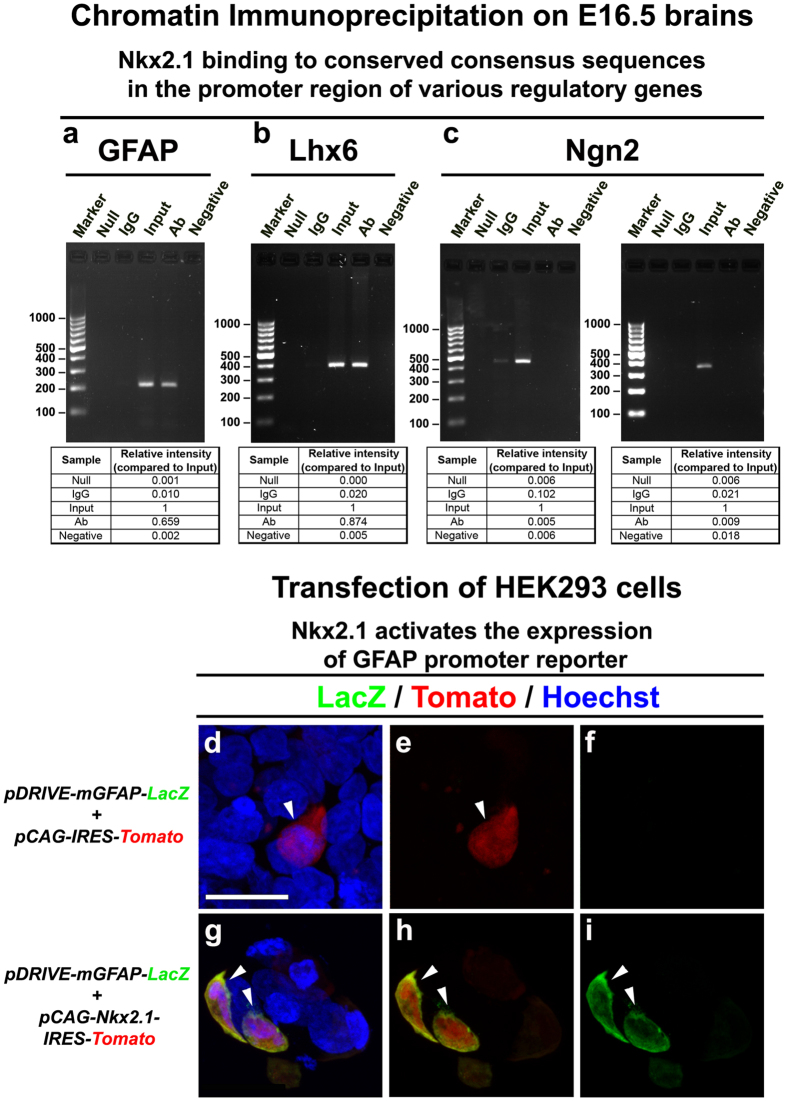
Binding of Nkx2.1 to conserved binding sequences in the promoters of various glial regulatory genes. Amplification of the putative Nkx2.1 binding sequences located in a 206 bp PCR product within the GFAP promoter **(a)** and in a 391 bp PCR fragment from the Lhx6 promoter **(b)** after chromatin immunoprecipitation (ChIP) with the Nkx2.1 antibody. Input DNA was added as the positive control as it contains the cross-linked sonicated genomic DNA taken before ChIP with the Nkx2.1 antibody; a strong signal was observed for all the promoter regions. No amplification of the Nkx2.1 core sequence (tcaag) located in two, 410 bp and 352 bp, PCR products from the Ngn2 promoter was detected **(c)**. No signal was detected in the negative control where no antibody was added for ChIP nor when no DNA was added while performing the PCR **(a–c)**. A very faint signal was detected in some samples immunoprecipitated with non-specific control IgG. These results suggest that Nkx2.1 binds *in vivo* to the promoter region of GFAP at putative Nkx2.1 binding sequences. The relative intensities of all the bands were calculated by assigning an arbitrary value of 1 to the input band. The quantifications are indicated below the gels. The Figure represents one of three independently performed assays. As a control, human embryonic kidney 293 (HEK293) cells were co-transfected with two reporter-constructs, namely, the *pDRIVE-mGFAP-LacZ* and the *pCAG-IRES-Tomato* plasmids **(d–f)**. Cell nuclei were counterstained in blue with Hoechst (**d**). In the control, only 4.26% of Tomato^+^cells were LacZ^+^, n = 94. To test the binding of Nkx2.1 to the GFAP promoter, HEK293 cells were co-transfected with two reporter constructs, namely, the *pDRIVE-mGFAP-LacZ* and the *pCAG-Nkx2.1-IRES-Tomato* plasmids **(g–i)**. Cell nuclei were counterstained in blue with Hoechst (**g**). Activation of the LacZ reporter was seen upon addition of the Nkx2.1 expression vector, thus confirming that Nkx2.1 activates the GFAP promoter, most probably by binding the sequence identified as Nkx2.1 consensus. Here, 98% of Tomato^+^cells were LacZ^+^, n = 50. Bar = 50 μm.

From our results, astroglial cell populations of the embryonic telencephalon are derived from Nkx2.1^+^ progenitors and Nkx2.1 regulates astrocyte precursor cell proliferation. We looked if the transcription factor Nkx2.1 binds to the promoter of the GFAP gene expressed in some brain astroglia and regulates its expression. Chromatin immunoprecipitation assays were performed on lysates of E16.5 embryonic brains. First we looked for DNA elements in the GFAP gene promoter and of a negative control gene, *Neurog2* that regulates dorsal precursors matching the consensus NK2 family binding sequence [GNNCACT(T/C)AAGT(A/G)(G/C)TT]^35^,^57^. Since a complete consensus binding sequence was not found, the core binding sequence T(C/T)AAG was chosen for analysis. As positive control, we used *Lhx6* (LIM homeodomain gene) for which the binding site of Nkx2.1 is already known^58^. Then, after shortlisting the position of putative Nkx2.1 binding sites, primers for the flanking sequences of all shortlisted binding sites (up to three) were made (see methods and Supp. Fig. S5A). We performed PCR on cross-linked and sonicated DNA pulled down using an anti-Nkx2.1 monoclonal antibody. The amplification of a putative Nkx2.1 binding sequence located within the *GFAP* gene (Fig. 9a) was found in the chromatin immunoprecipitated with the Nkx2.1 antibody, however, no positive interaction was detected for Neurog2 (Fig. 9c). Amplification of the already known Nkx2.1 binding site within the Lhx6 promoter was positive after immunoprecipitation with the anti-Nkx2.1 antibody (Fig. 9b). Furthermore, the PCR fragment(s) amplified in the GFAP promoter contained the core sequence CTCAAGT of the Nkx2.1 consensus binding sequence. This suggests that *in vivo*, Nkx2.1 binds to the promoter region of the astroglial GFAP regulatory gene at the highly conserved core-binding sequence of the consensus-binding site.

To investigate the influence of the binding of Nkx2.1 to this promoter sequence on the transcription of *GFAP* gene, we performed co-transfection studies in HEK293 cells. We used the expression plasmid *pDRIVE-mGFAP*, which contains the LacZ reporter under the control of the upstream −1679 bp mouse GFAP promoter sequence (that includes the putative Nkx2.1 binding site; Supp. Fig. S5B). This was co-transfected with the *pCAG-Nkx2.1-IRES-Tomato* plasmid that constitutively over-expresses Nkx2.1 and Tomato proteins under the control of the *pCAG* promoter. Co-transfection in HEK293 cells resulted in robust expression of the LacZ reporter (98% of Tomato^+^ cells were LacZ^+^, n = 50; Fig. 9g–i). Conversely, almost no expression was seen with co-transfection of the *pDRIVE-mGFAP* plasmid with a control *pCAG-IRES-Tomato* plasmid lacking the *Nkx2.1* cDNA (only 4.26% of Tomato^+^ cells were LacZ^+^, n = 94; Fig. 9d–f). This suggests that activation of the *GFAP* promoter fragment requires the presence of Nkx2.1, which probably acts at the binding sequence we identified.

Overall, the results show that the *Nkx2.1* homeobox gene regulates proliferation of astroglia that occupy the CC region during late embryonic stages.

## Discussion

Nkx2.1 is implicated in the specification of GABAergic interneurons and oligodendrocytes that occupy the embryonic telecephalon^3^,^16^,^37^,^41^,^42^,^43^. Recently, our group has shown that Nkx2.1 controls the production of the GABAergic interneurons, astrocytes and NG2 glia that populate the embryonic ventral telencephalon^18^,^44^. In this study, we now show that Nkx2.1 regulates the generation of the dorsal astroglia that populate the corpus callosum and its surrounding regions at late embryonic stages. Nkx2.1 controls astroglia by regulation of the proliferative capacity of Nkx2.1^+^ precursors present in the ventral progenitor regions, namely the MGE, the AEP/POA and the TS. By controlling the production of neurons and glia that populate the entire telencephalon, Nkx2.1 is a key factor for brain patterning in embryonic development.

### Origins and timing of gliogenesis in mouse brain

The exact timing of the generation and origin of embryonic glia is a topic of active investigation. Several mechanisms describing spatial and temporal differentiation of progenitors required for generation of the correct number of different types of glial cells have been proposed. Radial glia (RG) stem cells of the ventricular zone are proposed as the principal progenitor type during late embryonic brain development. During embryonic development, RG cells of the dorsal telencephalon not only generate most neurons of the cerebral cortex but also give rise later to two main glial sub-types, astrocytes and oligodendrocytes^19^,^22^,^23^,^26^,^29^. This hypothesis is supported by the generation of astrocytes and oligodendrocytes by spinal cord radial precursors^59^. Embryonic astroglia of the CC and the IG are also believed to originate from the radial glia of the dorsomedial pallium^30^,^31^. Contrary to what has been so far proposed, our study here shows for the first time that Nkx2.1 regulates the generation of transient midline dorsal embryonic astroglia of the CC and IG from the ventral progenitor regions. This is in accordance with our previous work showing that transient embryonic astroglia of the ventral telencephalon and transient embryonic NG2 glia occupying ventral and dorsal telencephalic regions are both derived from Nkx2.1^+^ progenitors from the subpallium^18^,^44^. Numerous studies also indicate that embryonic oligodendrocytes are produced in waves from the ventral telencephalic progenitors^16^,^17^. Additionally, subpopulations of telencephalic NG2 glia are documented to originate from ventral radial glia and pallial progenitors at E14^60^,^61^. Also, previous reports have shown that some postnatal astrocytes occupying the cerebral cortex, white matter and striatum originate from progenitor cells in the subventricular zone (SVZ) of the mammalian forebrain^33^. Recent evidence, however, shows that local populations of differentiated astrocytes constitute the primary source of postnatal glia and the SVZ progenitors contribute to only 3% of postnatal glia^34^.

The temporal competence of glial progenitors is not yet fully known. In the dorsal telencephalon, astrocyte gliogenesis was reported to occur only after neurogenesis (after E17 in mice) when the bipotential radial glial cells of the dorsal pallium differentiate into astrocytes^20^,^21^,^22^,^24^,^25^,^27^,^28^,^29^. The time of generation of some of the astrocytes occupying the CC midline region however is noted to be between E13 and postnatal day 2 (P2) with a peak at E14, much earlier than previously proposed^30^. Our previous work demonstrated that transient Nkx2.1-derived midline glia in the anterior commissure region are already generated by E14.5^18^. Here, we confirmed that transient Nkx2.1-derived astroglia in the CC and surrounding region are mainly generated between E14.5 to E16.5 too. Contrary to previous reports, we show that not only are these glia generated much earlier than previously proposed but also that they are generated from the ventral Nkx2.1^+^ progenitor regions. Hence, our study here sheds light upon both the spatial and temporal aspects of origin of embryonic telencephalic glia.

### Multilevel regulation of embryonic astrogliogenesis by Nkx2.1

The Nkx2.1-derived embryonic cell population is broadly divided into GABAergic neurons, astrocytes and NG2 glia based on their expression profiles. Only Nkx2.1^+^ astrocyte-like cells that are GLAST^+^ and/or GFAP^+^ maintain Nkx2.1 expression while other Nkx2.1-derived cells that are Olig2^+^ NG2 glia no longer express Nkx2.1 after differentiation. Loss of Nkx2.1 function leads to a drastic reduction in the numbers of astroglia and NG2 glia in the midline dorsal (CC, IG, MZG) and ventral telencephalic (mutant MGE* and POA*, and septum) regions. The loss of astroglia and NG2 glia is not accompanied by an increase in apoptotic cells indicating that loss is likely due to incapacity of the precursors to generate astroglia and NG2 glia. Indeed, further analyses revealed that the loss of glia is associated with a decrease in *Nkx2.1*-derived precursor division capacity. Accordingly, we observed a drastic decrease in GLAST^+^ precursors expressing the mut-Nkx2.1 in the VZ, SVZ of the mutant MGE*, mutant POA* and TS region, and in GLAST^+^ differentiated astrocytes expressing the mut-Nkx2.1 in the parenchyma (striatum, LPOA/LH, septum) in *Nkx2.1*^−/−^ compared to mice. The decreased presence of the precursors and differentiated astroglial population was accompanied by reduced proliferative status of the BrdU^+^ dividing cells containing mut-Nkx2.1 in the VZ and SVZ of mutant MGE*, mutant POA* and the septal nucleus region in *Nkx2.1*^−/−^ mice compared to control precursors. Additionally, *in vitro* differentiation of E14.5 *Nkx2.1*^−/−^ mutant MGE*, POA*-derived neurospheres revealed that progenitors were unable to generate GFAP^+^ astroglia expressing mut-Nkx2.1 though they still retained the capacity to generate post-mitotic neurons. Hence, the reduction in number of astroglia and NG2 glia can be attributed to inadequate proliferation of precursors in the three subpallial domains in the absence of Nkx2.1. Nonetheless, reduced generation of astroglia in the neurosphere assay could be due to decreased specification or differentiation capacity of precursors not expressing Nkx2.1. Evidence for this comes from previously published studies where loss of Nkx2.1 results in a ventral to dorsal re-specification of the MGE* to LGE*^37^. Likewise, we showed that in *Nkx2.1-Cre*^+^; *Rosa-DTA* mice, there is significant loss of differentiated astroglia at the anterior commissure midline without reduced precursor proliferation, thus, suggesting a role for Nkx2.1 after the proliferation step^18^. *Nkx2.1-Cre*^+^; *Rosa-DTA* mice cause the selective ablation of Nkx2.1-derived post-mitotic cells but not Nkx2.1^**+**^precursors due to a delay in expression and subsequent action of diphtheria toxin^18^. Also, the GLAST^+^ astroglia at the CC midline at E16.5-E18.5 still retain Nkx2.1 expression (Fig. 1c,d), pointing towards additional Nkx2.1 functions in mature astroglia. Performing a clonal analysis with the neurosphere assay where both the number and size of the astrocytic clones are estimated could provide further insights into Nkx2.1’s mode of action.

Chromatin immunopreciptation analysis performed in this study suggests that the Nkx2.1 might act by directly activating astroglial specific gene, such as GFAP as shown here. Future experiments where the putative Nkx2.1-binding site found in this study is mutated would further strengthen this result.

Thus, Nkx2.1 regulates the generation of dorsal telencephalic astroglia through multilevel controls that possibly involve mediating: (i) proliferation of Nkx2.1^+^ precursors, (ii) specification/differentiation of Nkx2.1^+^ precursors and (iii) transcriptional regulation of an astroglial gene.

### Nkx2.1 is important for several aspects of brain development in embryogenesis

In Nkx2.1 knockout mice several developmental abnormalities occur in the ventral forebrain^43^. Previous reports from our and other groups have shown that Nkx2.1 is not only important for regional specification of the ventral telencephalic regions, MGE and POA, but is also essential for the generation of a wide spectrum of Nkx2.1-derived lineages, including GABAergic interneurons, NG2 glia (or oligodendrocytes) and astrocytes that populate both the dorsal and ventral telencephalon from E12.5^3^,^14^,^16^,^17^,^18^,^37^,^42^,^43^,^44^,^54^,^58^,^62^,^63^,^64^. We showed previously that the absence of NG2 glia drastically affects the vascular development in all the telencephalon leading to severe blood vessels reduction of ramifications and connections^44^. We also demonstrated that a drastic reduction in Nkx2.1-derived cell populations leads to AC commissure agenesis and commissural axon misrouting^18^. Interestingly, the timing of generation of the Nkx2.1-derived cells precedes the arrival of the commissural axons at the AC midline, and some of the precursors in the TS and astrocytes surrounding the AC use *Slit2* to tunnel the axons through the anterior commissure^18^.

Also, the reduced GABAergic neuronal population and possibly the loss of astrocytes in the *Nkx2.1*^−/−^ mice lead to slight callosal axon branching and outgrowth defects in the CC tract^65^. Though *Nkx2.1*^−/−^ mice display a drastic loss of astroglia within CC and its surrounding regions including IG and MZG, the mice do not present CC agenesis and severe callosal axon bundles defects. Hence, these results show that these astroglia do not play a major role in CC axon guidance. These results contradict previously published reports where IG astroglia have been proposed to act as guidepost cells for callosal axons^31^,^66^. However, we cannot exclude the proposed contribution of GW radial glia in callosal axon guidance^66^,^67^,^68^ since they are not Nkx2.1-derived and hence, are not ablated in *Nkx2.1*^−/−^ mice. Thus, radial glia precursors occupying the GW and secreting Slit2 may be key player cells for callosal axon guidance during embryonic development.

Altogether, Nkx2.1 is able to perform its vast range of roles through regulation of proliferation of Nkx2.1^+^ precursors as shown here, and also possibly through an effect on glial specification/differentiation and survival. It appears that Nkx2.1 mediates part of these effects through transcriptional regulation, such as that shown for astroglial gene GFAP here. Interestingly, Nkx2.1 has previously been proposed to regulate several transcriptional programs that are important in vertebrate lung morphogenesis^69^. Notably, transcriptional regulation by Nkx2.1 in early (E11.5) and late (E19.5) mouse lung development^40^. Interestingly, in mouse lungs, Nkx2.1 also directly regulates cell cycle effectors and its loss alters cell cycle progression^40^. Further investigation into the complete repertoire of transcriptional regulation exerted by Nkx2.1 could provide interesting insights into Nkx2.1 mode of action and astroglial generation in the brain.

Along the same line, previous reports have shown that Nkx2.1 regulates the transcription of many thyroid-specific genes^35^,^36^,^37^ and activates pulmonary-surfactant^38^, as well as pituitary gland genes^39^. Moreover, it has been shown *in vitro*, that Nestin might be a target of *Nkx2.1*^70^. Hence, it is probable that Nkx2.1 displays functional conservation in brain, thyroid, pituitary, and lung.

Complex cellular and molecular interactions between glia, neurons and guidance cues produced by them, govern the formation of the midline structures such as the corpus callosum and anterior commissure. Several Nkx2.1-derived glial and neuronal populations populate these structures, and further understanding of the mode of regulation mediated by Nkx2.1 can help to understand the formation of dorsal and ventral telencephalic regions.

## Methods

### Animals

All studies on mice of either sex were performed in compliance with the national and international guidelines, and with the approval of the Federation of Swiss cantonal Veterinary Officers (authorization number 2164). Mice were housed under conditions of controlled temperature and illumination (12-hour light-dark cycle, with lights on at 07:00 am and off at 07:00 pm). Animals had *ad libitum* access to food and water and were monitored regularly. For staging of embryos, midday of the day of vaginal plug formation was considered as embryonic day 0.5 (E0.5). Wild-type mice maintained in a CD-1/SWISS genetic background were used for developmental analysis of the CC. We used wild-type (+/+) and homozygous mutant *Nkx2.1* mice^37^,^43^,^71^, which are referred as *Nkx2.1*^+/+^ and *Nkx2.1*^−/−^ respectively, in this work. We used heterozygous *GAD67-GFP* knock-in mice, described as *Gad1-EGFP* knock-in mice^53^. *Gad1-EGFP* knock-in embryos can be recognized by their GFP fluorescence. PCR genotyping of these lines was performed as described previously^72^. We used *GLAST-Cre ERT*^*TM*^ (purchased from Jackson Laboratory: Tg(Slc1a3-cre/ERT)1Nat/J) transgenic mice that have a tamoxifen-inducible Cre recombinase fused to the ligand binding domain of the estrogen receptor under the control of the *Slc1a3* (solute carrier family 1 (glial high affinity glutamate transporter; *GLAST*) promoter. We used *Nkx2.1-cre*^64^ and *Cspg4-cre* (Jackson Laboratory: *B6;FVB-Tg(Cspg4-cre)1Akik/J*)^51^ transgenic mice that have been described previously. The reporter mouse *Rosa26R–Enhanced yellow fluorescent protein (EYFP*)^73^ was used to reliably express EYFP under the control of the Rosa26 promoter upon Cre-mediated recombination.

For the induction of CreERT, Tamoxifen (20 mg/ml, Sigma, St Louis, MO) was dissolved at 37 °C in 5 ml corn oil (Sigma, St Louis, MO) pre-heated to 42 °C for 30 minutes. A single dose of 4 mg (250–300 μl) was administered to pregnant females at E14.5 by oral gavaging.

### Immunocytochemistry

Embryos were collected after caesarean section and quickly killed by decapitation. Their brains were dissected out and fixed by immersion overnight in a solution of 4% paraformaldehyde in 0.1 M phosphate buffer (pH 7.4) at 4 °C. Brains were cryoprotected in a solution of 30% sucrose in 0.1 M phosphate buffer (pH 7.4), frozen and cut as 50 μm-thick coronal sections for immunostaining.

Mouse monoclonal antibodies were: BrdU (1/50; Monosan), Nestin (1/600; Pharmingen) and GFAP (1/500; Chemicon). Rat monoclonal antibody was: L1 (1/200; Chemicon). Rabbit polyclonal antibodies were: Calretinin (1/2000; Swant); GFAP (1/500; DAKO); GFP (1/500; Molecular Probes); NG2 (1/100; Chemicon); Nkx2.1 (1/2000; Biopat); Olig2 (1/500; Millipore); RFP (1/500; Labforce) and S100ß (1/2500; Swant). Guinea pig antibody was: GLAST (1/200; Chemicon). Chicken antibody was: GFP (1/500; Aves).

Fluorescence immunostaining was performed as follows: non-specific binding was blocked with 2% normal horse serum in PBS 1X solution with 0.3% Triton X-100 for preincubation and incubations. The primary antibodies were detected with Cy3-conjugated (Jackson ImmunoResearch laboratories, West Grove, PA) and Alexa488-, Alexa594- or Alexa647-conjugated antibodies (Molecular Probes, Eugene, OR) used at 1:300 dilution. Sections were counterstained with Hoechst 33258 (Molecular Probes), mounted on glass slides and covered in Mowiol 4-88 (Calbiochem, Bad Soden, Germany).

DAB immunostaining was performed as follows: Endogenous peroxidase reaction was quenched with 0.5% hydrogen peroxide in methanol and non-specific binding was blocked by adding 2% normal horse serum in Tris-buffered solutions containing 0.3% Triton X-100 for preincubation and incubations. The primary antibodies were detected with biotinylated secondary antibodies (Jackson ImmunoResearch, West Grove, PA) and the Vector-Elite ABC kit (Vector Laboratories, Burlingame, CA). The slices were mounted on glass slides, dried, dehydrated, and covered with Eukitt.

### BrdU tracing studies

To label cells in the S-phase of the cell cycle at the suitable embryonic stages (E12.5, E14.5 and E16.5), pregnant female mice were injected intraperitoneally with a solution of 8 mg/ml of 5-bromo-2′-deoxyuridine (BrdU; Sigma, St Louis, MO) in PBS (0.15 M NaCl, 0.1 M phosphate buffer, pH = 7.4) at a final concentration of 50 mg/kg body weight. To trace the division rate of the subpallial precursors, pregnant females were sacrificed 1-2 hours post-injection. To trace the date of genesis of the CC astrocytes, pregnant females were sacrificed when embryos were E18.5. BrdU was revealed by DAB or fluorescence immunostaining (as above) after a treatment with 2 M HCl for 30 min at room temperature.

### Imaging

DAB stained sections were imaged with a Zeiss Axioplan2 microscope equipped with 10×, 20×or 40× Plan neofluar objectives and coupled to a CCD camera (Axiocam MRc 1388 × 1040 pixels). Fluorescent-immunostained sections were imaged using confocal microscopes (Zeiss LSM 510 Meta, Leica SP5 or Zeiss LSM 710 Quasar) equipped with 10×, 20×, 40× oil Plan neofluar and 63 × oil, 100×oil Plan apochromat objectives. Fluorophore excitation and scanning were done with an Argon laser 458, 488, 514 nm (blue excitation for GFP and Alexa488), with a HeNe laser 543 nm (green excitation for Alexa 594 and CY3), with a HeNe laser 633 nm (excitation for Alexa 647 and CY5) and a Diode laser 405 nm (for Hoechst-stained sections). Z-stacks of 10–15 planes were acquired for each CC coronal section in a multitrack mode avoiding crosstalk.

All 3D Z stack reconstructions and image processing were performed with Imaris 7.2.1 software. To create real 3D data sets, we used the mode “Surpass”. The colocalization between two fluorochromes was calculated and visualized by creating a yellow channel. Figures were processed in Adobe Photoshop© CS4 and CS5 and schematic illustrations in Fig. 2 were produced using Adobe Illustrator© CS4.

### *In vivo* Quantifications

#### Glial cell population analysis

In 50 μm thick brain sections of *Nkx2.1*^+/+^ and *Nkx2.1*^−/−^ embryos at E18.5, the astroglial cells were labeled with GFAP and polydendroglial cells were labeled with NG2. Cells were counted in the CC, IG, MGE, MZG and POA regions from at least 4 brains per condition. Cell densities were reported per surface unit area (number of cells/mm^2^). The quantification was done using Neurolucida 9.0 and Neurolucida 9.0 Explorer© software.

In 50 μm thick brain sections of *Nkx2.1*^+/+^ and *Nkx2.1*^−/−^ embryos at E18.5, the astroglial cells that were labeled for Olig2 or both Olig2 and GLAST were counted in the CC mid from at least 2 brains per condition. Olig2 staining labeled the glial cell bodies while GLAST labeled both the cell bodies and processes. The cell densities were determined in the medial and lateral part of the CC. The cell densities were reported per volume unit (number of cells/mm^3^). The quantification was done using Imaris^®^ 7.2.1 software.

#### Nkx2.1^+^ and GLAST^+^ and BrdU^+^ proliferation analyses

Pregnant female mice were injected intraperitoneally with a solution of 8 mg/ml of 5-bromo-2′-deoxyuridine in PBS to a final concentration of 50 mg/kg body weight. To trace the division rate of the subpallial precursors, pregnant females were sacrificed 2 hours post-injection. Embryos were collected after caesarean section and quickly killed by decapitation. Their brains were dissected out and fixed by immersion overnight in a solution of 4% paraformaldehyde in 0.1 M phosphate buffer (pH 7.4) at 4 °C. In 50 μm thick brain sections of *Nkx2.1*^+/+^ and *Nkx2.1*^−/−^ embryos at E16.5, Nkx2.1^+^ cells, BrdU^+^ dividing cells and GLAST^+^ precursors or post-mitotic astroglial cells of the MGE, POA and TS were counted in the VZ, SVZ and in the parenchyma of each region, from at least 4 brains per condition. Nkx2.1 and BrdU staining labeled the cell bodies while GLAST labeled both the cell bodies and processes. Although the expression level of truncated Nkx2.1 protein (mut-Nkx2.1) in *Nkx2.1*^−/−^ embryos was low, it was easily identifiable using immunostaining and quantifications were performed on higher magnification images where the staining could be easily visualized. The percentage of *Nkx2.1*-derived dividing precursors or post-mitotic glial cells were determined as follows: In each sub region and for each condition, a sample of at least four different Z-stacks was acquired at 100x magnification using a Leica SP5 confocal microscope. The Z-stacks comprised 10 planes that were acquired in a multitrack mode avoiding any crosstalk. Thereafter, in order to exclude the possibility of quantifying the same cells more than once, snapshots of only 3 planes (from the acquired 10 planes), were analyzed. The quantification of Nkx2.1, BrdU, GLAST and Hoechst staining was done on each snapshot separately Imaris^®^ 7.2.1 software.

#### Cell death analysis

In brain sections of *Nkx2.1*^+/+^ and *Nkx2.1*^−/−^ embryos at E16.5, apoptotic cells labeled with either cleaved-caspase 3 or TUNEL were counted in the CC, MGE, SEP, and POA from at least 2 brains per condition. 50 μm thick brain sections were used for cleaved-caspase 3 staining whereas 10 μm thick brain sections were utilized for TUNEL staining. Cell nuclei were counterstained with Hoechst. For each condition, at least 5 different Z-stacks were obtained at 100x magnification using a Leica SP5 microscope. The number of apoptotic nuclei were counted and reported as an absolute number per section (the surface area of one section was 24119.332 μm^2^). The quantification was done using Neurolucida 9.0 and Neurolucida 9.0 Explorer© software.

### Neurosphere generation and microscopic analysis

The protocol has been adapted from Arsenijevic *et al*., 2001.

#### Primary culture and sphere passaging

The brains of embryos at developmental stage E14.5 were collected as described above. They were carefully removed from the skull into ice-cold sterile dissecting medium (MEM 1X) complemented with Glucose 1 M (5 ml/100 ml). Thereafter, the brains were embedded in low melting point Agarose 3% (LMP-Agar, Gibco) at 37 °C, and cut into 250 μm thick slices using a vibratome (Leica© VT 1000 S). The sections were collected in the ice-cold dissecting medium. The areas of interest (MGE, POA and SEP) were dissected out using two tungsten needles under a stereomicroscope (Leica© MZ16F). The dissected pieces of tissue were then collected into 1 ml ice-cold sterile Hormone Mix Medium (MHM 1X) supplemented with Penicillin (50 U/ml) and Streptomycin (50 U/ml) (GIBCO). The Hormone Mix Medium is a growing medium containing DMEM and F-12 nutrient (1:1), glucose (0.6%), glutamine (2 mM), sodium bicarbonate (3 mM), HEPES buffer (5 mM), transferrin (100 mg/ml), insulin (25 μg/ml), progesterone (20 nM), putrescine (60 μM), selenium chloride (30 nM)^74^. Brain tissue pieces were mechanically dissociated in sterile conditions with a fire-polished pipette in the Hormone Mix Medium. The pipette was rinsed before the dissociation of each new region.

The dissociated cells were then grown in Hormone Mix Medium complemented with Pen/Strep and EGF in 6-well dishes (Nunclon Surface, NUNC Brand Products, Nalge Nunc International) at a concentration of around 10^4^–10^5^ cells per 1 ml and 4 ml per dish. After 6-7 days *in vitro* (DIV) at 37 °C in a 5% CO_2_ atmosphere, the sphere cultures were expanded. Primary spheres were dissociated mechanically and cells were plated at the density of 2 × 10^6^ cells for 40 ml in a flask (Nunclon Surface, NUNC Brand Products, Nalge Nunc International). Sphere passages were done every 7 DIV, by sphere dissociation and transfer of 2 × 10^6^ cells to a new 40 ml flask.

#### Differentiation of spheres

After 7 DIV, the neurospheres of optimum size were chosen under a steremicroscope (Nikon©) and transferred individually and plated onto poly-L-ornithine coated coverslips in 24-well plates (Nunclon Surface, NUNC Brand Products, Nalge Nunc International). Each coverslip contained about ten spheres and 1 ml of Hormone Mix Medium supplemented with Pen/Strep and 2% fetal bovine serum (FBS).

#### Immunofluorescence on differrentiated Neurospheres

After 7 DIV, the neurospheres were fixed in 4% PFA for 20 minutes and permeabilized with 0.3% triton/PBS1X for 3 minutes. Coverslips were incubated with primary antibodies diluted in PBS containing 10% NHS for 2 hours at room temperature, followed by secondary fluorescent antibodies for 45 minutes at 37° and Hoechst staining for 5 minutes.

#### Neurosphere production analysis

MGE- and POA-derived neurospheres were obtained from Nkx2.1^+/+^ and Nkx2.1^−/−^ E14.5 embryos. After 7 DIV, the neurospheres were differentiated and immunostained as mentioned above. Two different brains were used for each condition and were labeled for Nkx2.1, GFAP, GLAST, and βIII tubulin. Cell nuclei were counterstained with Hoechst. For each condition, a total of at least 5 different Z-stacks in 5 different neurospheres were acquired at 100x magnification using a Leica SP5 microscope. The percentage of Nkx2.1^+^/GFAP^+^ differentiated astrocytes and Nkx2.1^+^/βIII tubulin^+^ differentiated neurons were counted directly on the Z-stacks by using Imaris^®^ 7.2.1 software.

### Chromatin Immunoprecipitation

Chromatin immunoprecipitation was conducted on E16.5 brain samples according to the instructions provided by the manufacturer (Upstate, 17-295), using 2 μg of mouse anti-Nkx2.1 monoclonal antibody (MS699-P, Lab Vision). For crosslinking, 1% PFA was used. For sonication, six bursts of 45 seconds ON (30% power) and 30 second OFF were given and samples were kept on ice throughout. Mouse Genome Assembly data mm9 was used to map sites.

A 391 bp PCR fragment of the *Lhx6*promoter that includes a Nkx2.1 binding sequence at position −240 bp relative to the putative transcriptional start site was identified using primers 5′-tttgtaccgagagtaggagaagg and 5′-gtcctaactttgtagtgggcattt.

A 206 bp PCR fragment of the *GFAP*promoter that includes a putative Nkx2.1 binding sequence (ctcaagt) at position −838 bp relative to the putative transcriptional start site was found to be a binding target and was identified using primers 5′- tggataagaggccacagagg and 5′- cctctcccctgaatctctcc.

Primers against two fragments of the *Neurogenin2* promoter region, comprising the core Nkx2.1 binding consensus sequence (tcaag), were made. 1) Primers 5′-cgggattctgactctcactaattc and 5′-aatggttctaaagctcctgttgg were designed to amplify a 410 bp PCR fragment with the core consensus Nkx2.1 binding sequence at position −668 bp relative to the putative transcriptional start site. 2) Primers 5′-cgggattctgactctcactaattc and 5′-aatggttctaaagctcctgttgg were designed to amplify another 352 bp PCR fragment with the core consensus Nkx2.1 binding sequence at position −4073 bp relative to the putative transcriptional start site.

Following controls were used: (1) Null – beads only without any antibody, (2) IgG – beads with an isotype matched control immunoglobulin (Ig) to know the background of the assay, (3) Input – starting material taken before immunoprecipitation with antibody, and (4) Negative – non-template control used for the PCR reaction to spot any contamination.

For quantification, ImageJ was used to measure the band densities (https://imagej.nih.gov/ij/docs/menus/analyze.html#gels). The relative intensities of all the bands (null control, IgG control, immunoprecipitated with antibody, and negative control) were calculated by assigning an arbitrary value of 1 to the input band.

### Transfection of HEK293 cells

A suspension of HEK293 cells adapted to serum-free growth medium was plated at 1 × 10^6^ cells in 4 ml media in a 60mm plate. For formation of the transfection complexes, a 3:1 ratio of FuGENE^®^ HD Transfection Reagent (μl): plasmid DNA (μg) was prepared and used for transfection. The study was performed by co-transfecting an expression plasmid for constitutive over-expression of Nkx2.1 (*pCAG-Nkx2.1-IRES-Tomato*) or a control plasmid (*pCAG-IRES-Tomato*) with the *pDRIVE-mGFAP* plasmid containing the GFAP promoter region in front of the LacZ reporter gene. The pCAG promoter is constructed of following sequences: (**C**) cytomegalovirus early enhancer element, (**A**) promoter, the first exon and intron of the chicken beta-actin gene and (**G**) the splice acceptor of the rabbit beta-globin gene. The mouse GFAP promoter sequence from the *pDRIVE-mGFAP* plasmid (#pdrive-mgfap, InvivoGen) used for transfection experiments is shown in Supp. Fig. S5B. Transfection complexes were formed by mixing 2 μg of each of the two plasmids with 12 μl of Fugene transfection reagent and 188 μl of Optimem reduced serum media. The mix was incubated at room temperature for 20 minutes and then added to the cell plates. The cell plates were kept in the 37 °C incubator and gene expression analysis was done after 24–48 hours of transfection. Fluorescence immunostaining was done to visualize the presence and level of *LacZ* expression. Tomato signal was visible by direct fluorescence, however, for a clearer visualization of Tomato signal anti-RFP immunostaining was done (described above).

### Experimental design and statistical analysis

For all analyses at least three independent experiments were performed. Mutant embryos were always compared with controls originating from the same litter. Qualitative, morphological and cellular change phenotypes of the control and mutant brains were ascertained blindly. Quantitative analyses were verified blindly by a second experimenter. The results from all quantifications were analyzed with the aid of Statview software (SAS Institute Inc.). For all analyses, values were first tested for normality and the variance of independent populations were tested for equality. Values that followed a normal distribution were compared using Student’s *t*-test. To show the degree of significance for quantitation included in the study, we added the number of asterisks based on the following standard p-value criteria: ***p < 0.001; **p < 0.01; *p < 0.05.

### Atlas and nomenclature

The neuroanatomical nomenclature is based on the “Atlas of the prenatal mouse brain”^75^.

## Additional Information

**How to cite this article:** Minocha, S. *et al*. Nkx2.1 regulates the generation of telencephalic astrocytes during embryonic development. *Sci. Rep.* **7**, 43093; doi: 10.1038/srep43093 (2017).

**Publisher's note:** Springer Nature remains neutral with regard to jurisdictional claims in published maps and institutional affiliations.

## Supplementary Material

Supplementary Figures and Legends
