# Supplementary material for: Nkx2.1 regulates the generation of telencephalic astrocytes during embryonic development
Source: Sci Rep. 2017 Mar 7;7:43093. doi: 10.1038/srep43093 (PMC5339799; doi:10.1038/srep43093)
Supplement: Supplementary Figures and Legends [file srep43093-s1.pdf]

Supplementary information

Nkx2.1 regulates the generation of telencephalic astrocytes during embryonic development

Shilpi Minocha[1*], Delphine Valloton[1*], Yvan Arsenijevic[2], Jean-René Cardinaux[3], Raffaella Guidi[3], Jean-Pierre Hornung[1], and Cécile Lebrand[1, ✍].

[1] Department of Fundamental Neurosciences, University of Lausanne, Rue du Bugnon 9, CH-1005 Lausanne, Switzerland.

[2] Department of Ophthalmology, University of Lausanne, Hôpital ophtalmique Jules-Gonin, Av. de France 15, CH-1004 Lausanne, Switzerland.

[3] Department of Psychiatry, Center for Psychiatric Neuroscience, Lausanne University Hospital, Prilly, CH-1008 Lausanne, Switzerland.

✍: Correspondence to Dr. Cécile Lebrand, Department of Fundamental Neurosciences, University of Lausanne, Rue du Bugnon 9, 1005 Lausanne, Switzerland. Telephone: +41 21 692 51 30. Fax: +41 21 692 51 05.

Email: cecile.lebrand@unil.ch.

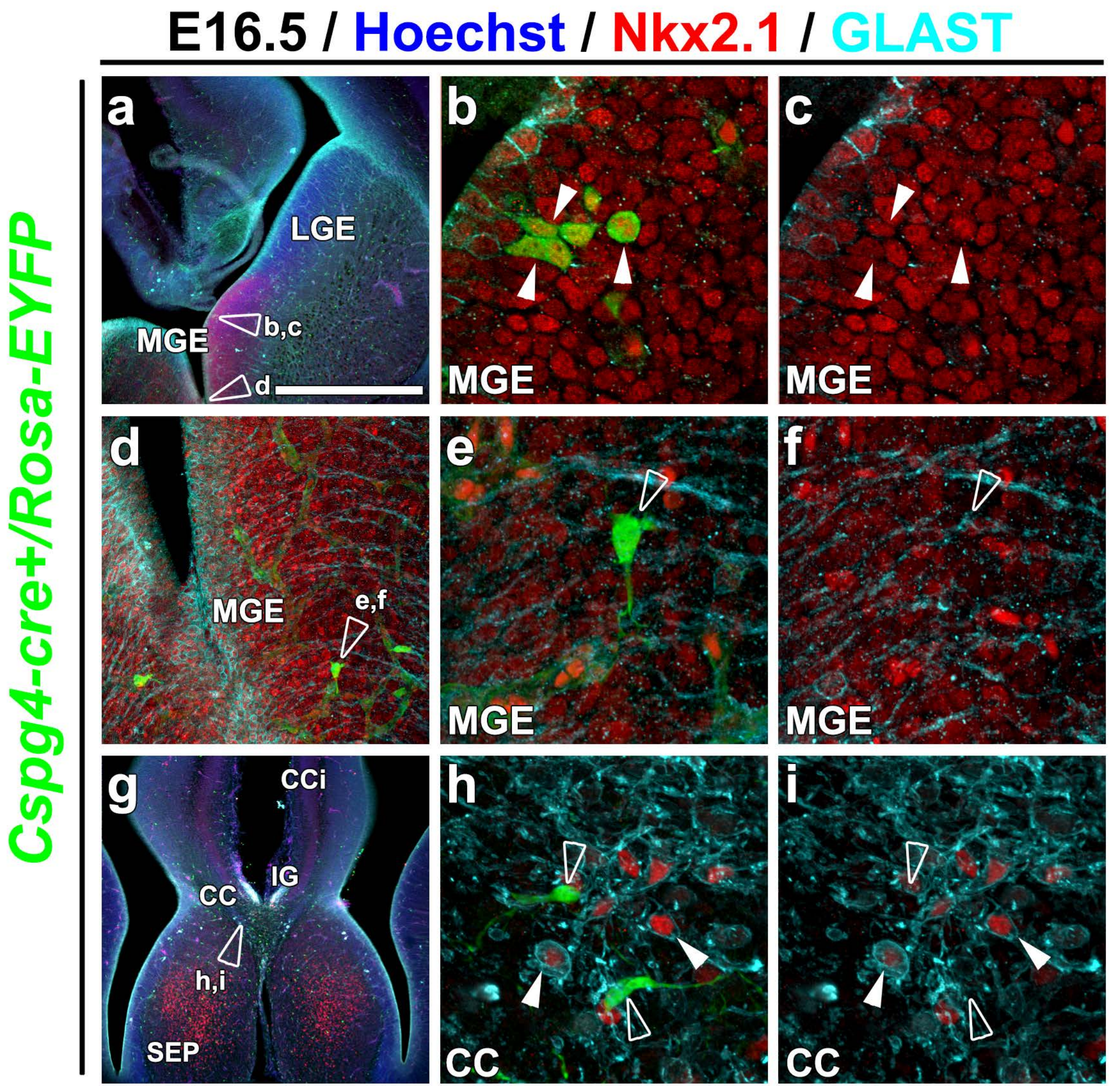

E16.5 / Hoechst / Nkx2.1 / GLAST
Cspg4-cre+/Rosa-EYFP
a
LGE
MGE
b,c
d
b
MGE
c
MGE
d
MGE
e,f
e
MGE
f
MGE
g
CCi
IG
CC
h,i
SEP
h
CC
i
CC

Supplementary Figure S1. Fate-mapping study of *Nkx2.1*-regulated NG2$^+$ glia using the *Cspg4-Cre$^+$/Rosa-EYFP* reporter mice.

(a-i) Triple immunohistochemistry for the EYFP, Nkx2.1 and GLAST on coronal sections from *Cspg4-Cre$^+$/Rosa-EYFP* mice (n=3) at E16.5. Cell nuclei were counterstained in blue with Hoechst **(a** and **g)**. **b, c, e, f, h** and **i** are higher power views of the regions shown in **a, d** and **g**, respectively. At E16.5, NG2$^+$ (or Cspg4$^+$) glia visualized by the EYFP signal were found to originate from Nkx2.1$^+$ subpallial sites such as the MGE **(a-c** and **d-f)**. The colocalization between Nkx2.1 (in red) and the EYFP signal (in green) is observed in few cells in the SVZ of the MGE (solid arrowheads in **b** and **c**) but as soon as the NG2$^+$ cells start to differentiate and migrate, Nkx2.1 is down-regulated and is no anymore more visible (open arrowheads in **e-f** and **h-i**). By contrast, Nkx2.1 is still expressed in GLAST$^+$ astroglial cells within the CC midline (solid arrowheads in **h-i**).

(CC) corpus callosum; **(CCi)** cingulate cortex; **(CI)** cingulate bundle; **(IG)** induseum griseum; **(MGE)** medial ganglionic eminence; **(SEP)** septum.

Bar = 675 μm in **a** and **g**; 160 μm in **d**, 40 μm in **b, c, e, f, h** and **i**.

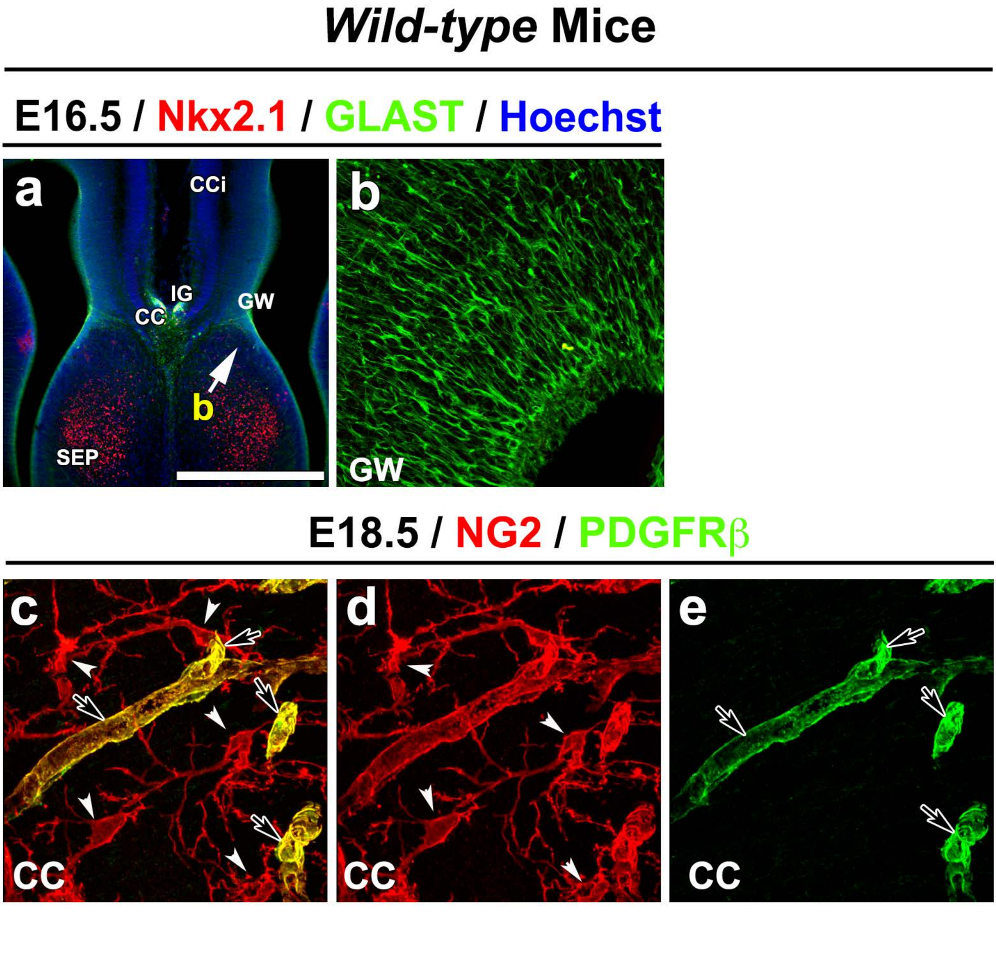

Wild-type Mice
E16.5 / Nkx2.1 / GLAST / Hoechst
a
CCi
IG
GW
CC
b
SEP
b
GW
E18.5 / NG2 / PDGFRβ
c
CC
d
CC
e
CC

Supplementary Figure S2. Radial glial cells within glial wedge region are Nkx2.1-negative and NG2 immunostaining can be used to clearly identify the NG2 glia

(a-b) Double immunostaining for Nkx2.1 and GLAST on coronal sections from WT mice (n=3) at E16.5. Cell nuclei were counterstained in blue with Hoechst **(a)**. **b** is higher power view of the regions shown in **a**. The $GLAST^{+}$ radial glial cells within glial wedge **(GW)** do not express Nkx2.1.

(c-e) Double immunostaining for NG2 and PDGFRβ on coronal sections from WT mice (n=3) at E18.5. Though $PDGFR\beta^{+}$ pericytes adjacent to the vessels are $NG2^{+}$ (arrows), they can be clearly differentiated from NG2 glia (white arrowhead) due to substantial difference in morphology.

(CC) corpus callosum; **(CCi)** cingulate cortex; **(GW)** glial wedge; **(IG)** induseum griseum; **(SEP)** septum.

Bar = 675 μm in **a** and 40 μm in **b, c-e.**

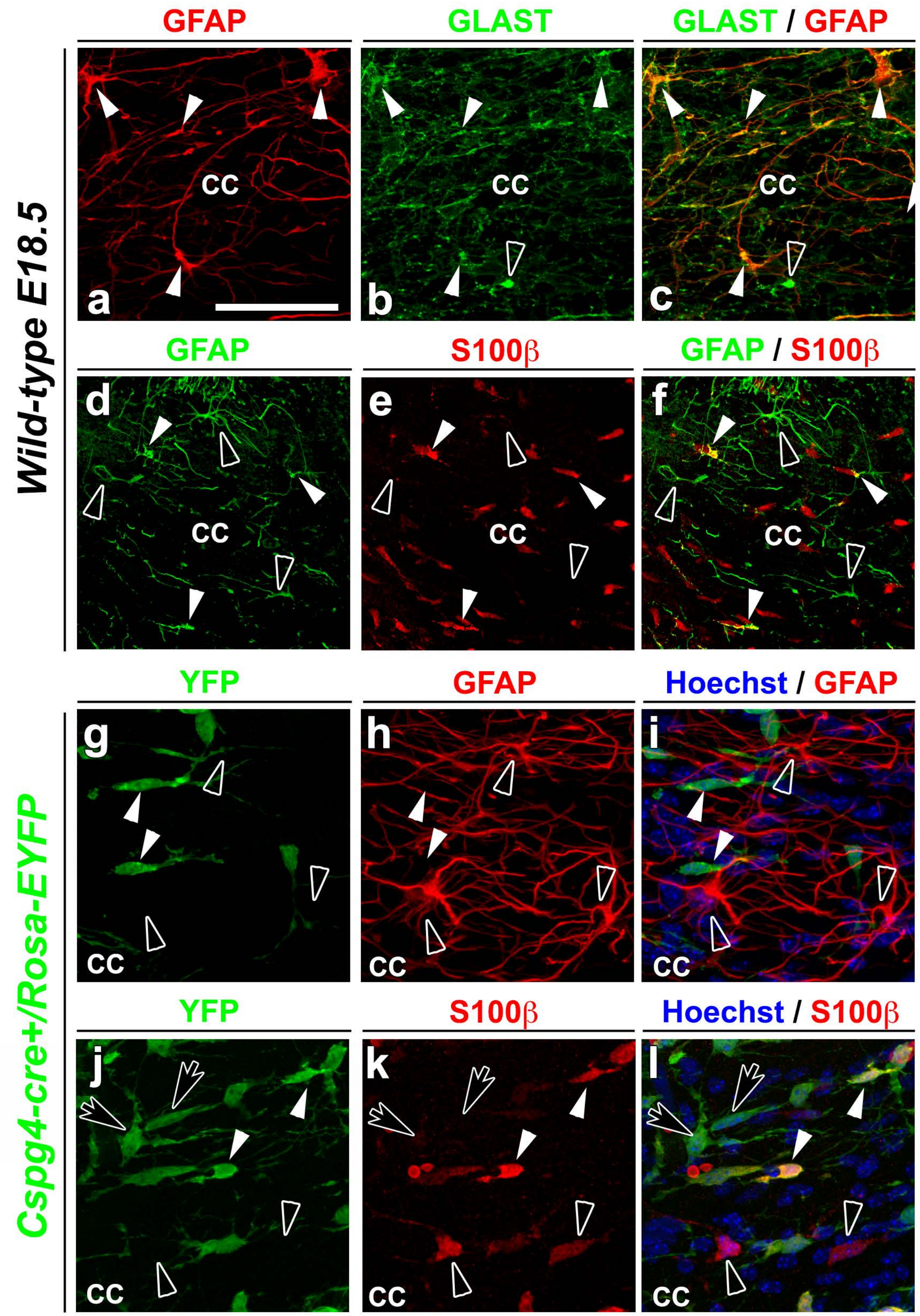

GFAP
GLAST
GLAST / GFAP
GFAP
S100β
GFAP / S100β
YFP
GFAP
Hoechst / GFAP
YFP
S100β
Hoechst / S100β
Wild-type E18.5
Cspg4-cre+/Rosa-EYFP
cc
a
b
c
d
e
f
g
h
i
j
k
l

Supplementary Figure S3. Characterization of glial subtypes in mouse embryonic brain.

(a-f) Double immunostaining for GFAP and GLAST (a-c) (n=3) and GFAP and S100β (d-f) (n=3) on coronal sections from WT mice (n=3) at E18.5. **(a-c)** The GFAP$^+$ glia co-express GLAST (closed arrowheads) whereas some glia are positive for GLAST alone (open arrowhead). **(d-f)** Some GFAP$^+$ glia co-express S100β (closed arrowheads) while several others do not express S100β (open arrowheads). Also, several S100β$^+$ cells do not express GFAP.

(g-l) Double immunostaining for YFP and GFAP (g-i) (n=3) and YFP and S100β (j-l) (n=3) on coronal sections of *Cspg4-cre*$^+$*/Rosa-EYFP* mice (n=3) at E18.5. **(g-i)** The NG2-derived glia, depicted by YFP staining (white arrowheads), do not express GFAP. The GFAP$^+$ glia (open arrowheads) and NG2$^+$ glia (closed arrowheads) are two distinct mutually exclusive populations. **(j-l)** Some NG2-derived glia co-express S100β (closed arrowheads) while several others do not express S100β (open arrows). Also, several S100β$^+$ cells are not part of the NG2-derived population (open arrowheads).

(CC) corpus callosum.

Bar = 40 μm.

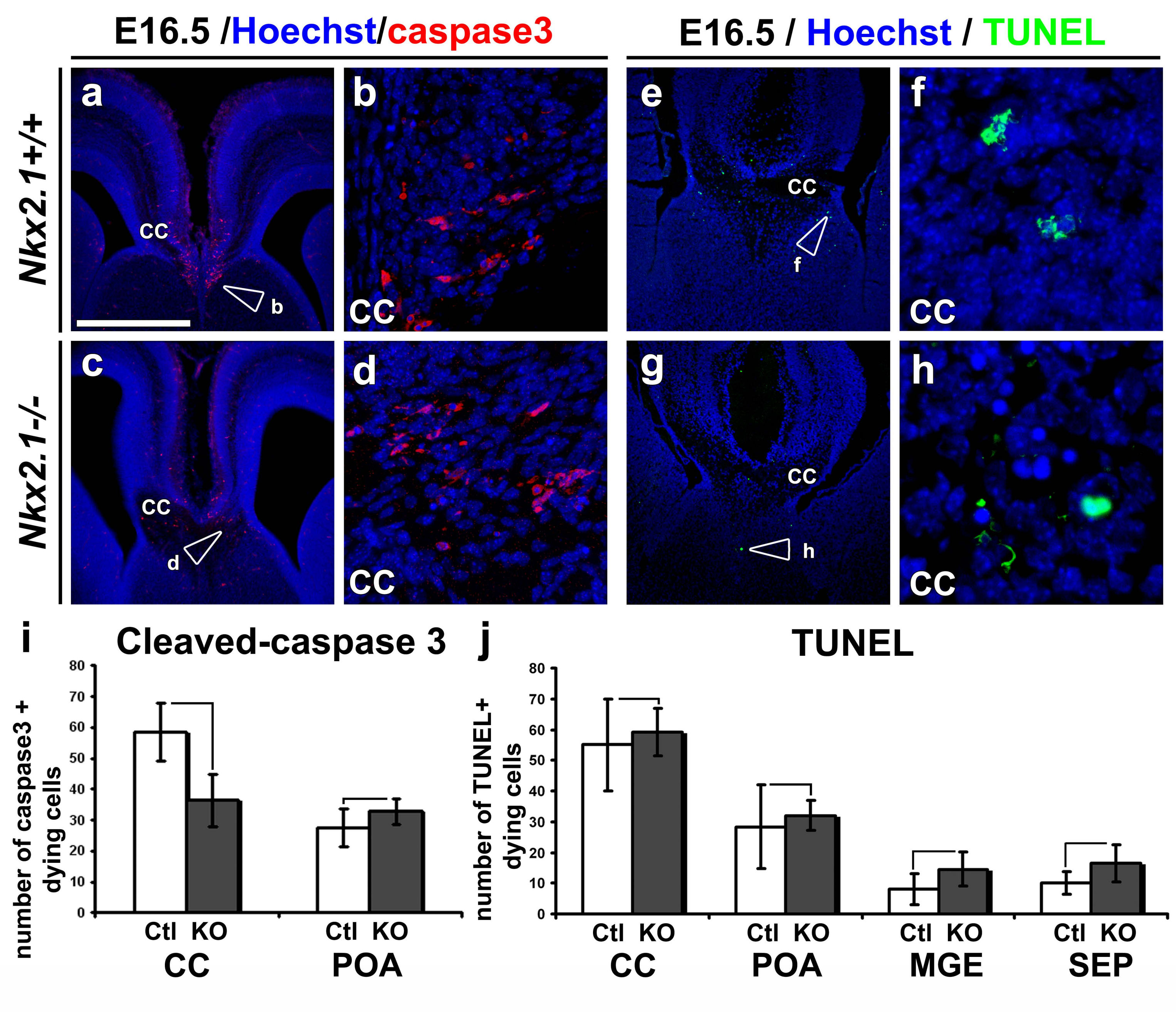
E16.5 /Hoechst/caspase3
E16.5 / Hoechst / TUNEL
Nkx2.1+/+
Nkx2.1-/-
a
b
c
d
e
f
g
h
CC
i
Cleaved-caspase 3
number of caspase3 + dying cells
Ctl KO
CC
POA
j
TUNEL
number of TUNEL+ dying cells
Ctl KO
CC
POA
MGE
SEP

Supplementary Figure S4. ***Nkx2.1***$^{-/-}$ **mice brains do not show any increase in cell death at E16.5.**

(a-d) Single immunohistochemical staining for the cleaved-caspase 3 (n=4 for CC region and n= 5 for POA region in *Nkx2.1*$^{+/+}$ or *Nkx2.1*$^{+/-}$ controls (Ctl); n=6 for CC region and n=10 for POA region in *Nkx2.1*$^{-/-}$ mice) and **(e-h)** TUNEL staining (n=16 for CC in Ctl mice, n=22 for CC in *Nkx2.1*$^{-/-}$ mice; n=6 for POA in Ctl mice, n=5 for POA in *Nkx2.1*$^{-/-}$ mice; n=10 for MGE in Ctl mice, n= 11 for MGE in *Nkx2.1*$^{-/-}$ mice; n=7 for SEP in Ctl mice, n=14 for SEP in *Nkx2.1*$^{-/-}$ mice) on CC coronal sections from Ctl **(a-b** and **e-f)** and *Nkx2.1*$^{-/-}$ mice **(c-d** and **g-h)** at E16.5. Cell nuclei were counterstained in blue with Hoechst. **b**, **d**, **f** and **h** are higher magnified views of the CC region seen in **a**, **c**, **e** and **g**, respectively. **(i** and **j)** Bars (mean ± SEM from a sample of n=4-16 sections in the Ctl and n=5-22 sections in *Nkx2.1*$^{-/-}$ mice depending on the region studied) represent the number of dying cells labelled by the cleaved-caspase 3 or by the TUNEL staining and displaying pyknotic nuclei per section (surface area/section=24119.332 mm^2), in the CC, POA, MGE and SEP of *Nkx2.1*$^{-/-}$ (KO) compared to *Nkx2.1*$^{+/+}$ or *Nkx2.1*$^{+/-}$ controls (Ctl). No significant differences were observed in the number of dying cells in *Nkx2.1*$^{-/-}$ mice brains compared to the Ctl. p-value= 0.1225 for CC and 0.4618 for POA with cleaved caspase 3 staining. p-value= 0.7934 for CC, 0.8193 for POA, 0.4032 for MGE, and 0.4879 for SEP with TUNEL staining.

(CC) corpus callosum; **(MGE)** medial ganglionic eminence; **(POA)** preoptic area; **(SEP)** septum.

Bar = 675 μm in **a**, **c**, **e** and **g**; 60 μm in **b** and **d**; 40 μm in **f** and **h**.

A.

ataaaagcagcccacggggctgcccttgccatatgcctcactggcggcag

agaacaaggctctattcagcgagtaccctggagtagacaccagaagccca

agcatgggcagaggaaggcaggggttgggggagcagagctgtctgtgtt

ccagaagcccaaggacacagatggctaaggcgcctgggagagggacctga

gtggaagagatagatgggcctgaagtctcaagcagcaacagcctcctccc

cgccattggtgagggtggggtttggtttcccggacctacatatccctcag

aggcctggtgtgtaggaatttaaaggggtaaatctcctgagagaatgag

gggtacccaggaagacggggtgttacagaaagaaagactccagcatgcac

agccaactcattcaaaactactctgtcaggggctgccaggggccaggctc

ggggtgggggtgggggcaacgagaagctggatcagggagaaatggccc

actaggctggataagaggccacagaggggctcaggaatgaagcctgctgt

cttaccctattaggatctgcgtgcataccttctgccgtgcactctaaaca

cacagccagagg**ctcaagt**tgaccctggagtcacagagagggctccaacc

ttagccctccactcctgaactccaggaatgagaagatagagttggagaga

ttcaggggagaggactctgttgagaatgggggtcacaggaaactgtaata

taggttgatcccggaggaagggaataggttcttcaagttcctagcatctc

acaggcccccagagaaggacagagttggggtggtcctggcttacaggctc

taagaactggaagctgattaccccaccgagctgtgcactctctgtctctg

tctctgtgtgtgcgctcgtgcacacttatcacacaaatgttcatgtgtgt

gcacatacatgtgttgagaccagaggtcaacctcaggcactgttgccttg

gttttctgagagagcatttctctctggatctggaactcgccaattagtga

gagccaggaagtctgctgattttcactgcccagcactggagtttacaagt

atgcactgtcaacccaggccttttgtattcattctgcagctagaacttgg

gtgggtcttcatgcttgacaggcaagcaatttatggactaagctgttccc

tcggccctctcttgacccatttaccagaaaggggggttccttgatcaatgg

cgaagccaggctggtgttcccaagaaagccttgactctgggtacagtgac

ctcagtggggtgagaggagttctccccctagctgggctggggcccagctc

caccccctcaggctattcaatgggggtgcttccaggaagtcaggggcaga

tttagtccaacccgttcctccataaaggccctgacatcccaggagccagc

ATGGAGCGGAGACGCA

B.

gtctgtaagctgaagacctggcagtgctgagctggtcagcccccaggacctccttttgtgcccacgagtgaatca

ccttggcatagacataatggtcaggggtgggcacgcagcctgcttcccgctgtgctccaggcctccttcgatgct

ttccgagaagtctattgagctgggagcttgtactgcacccggggctgacatcctggcatcctgggataaaagcag

cccacggggctgcccttgccatatgcctcactggcggcagagaacaaggctctattcagcgagtaccctggagta

gacaccagaagcccaagcatgggcagaggaaggcaggggttgggggagcagagctgtctgtgttccagaagccc

aaggacacagatggctaaggcgcctgggagagggacctgagtggaagagatagatgggcctgaagtctcaagcag

caacagcctcctccccgccattggtgagggtggggtttggtttcccggacctacatatccctcagaggcctggtg

tgtaggaatttaaaggggtaaatctcctgagagaatgaggggtacccaggaagacggggtgttacagaaagaaa

gactccagcatgcacagccaactcattcaaaactactctgtcaggggctgccaggggccaggctcggggtggggg

gtgggggcaacgagaagctggatcagggagaaatggcccactaggctggataagaggccacagaggggctcagg

aatgaagcctgctgtcttaccctattaggatctgcgtgcataccttctgccgtgcactctaaacacacagccaga

gg**ctcaagt**tgaccctggagtcacagagagggctccaaccttagccctccactcctgaactccaggaatgagaag

atagagttggagagattcaggggagaggactctgttgagaatgggggtcacaggaaactgtaatataggttgatc

ccggaggaagggaataggttcttcaagttcctagcatctcacaggcccccagagaaggacagagttggggtggtc

ctggcttacaggctctaagaactggaagctgattaccccaccgagctgtgcactctctgtctctgtctctgtgtg

tgcgctcgtgcacacttatcacacaaatgttcatgtgtgtgcacatacatgtgttgagaccagaggtcaacctca

ggcactgttgccttggttttctgagagagcatttctctctggatctggaactcgccaattagtgagagccaggaa

gtctgctgattttcactgcccagcactggagtttacaagtatgcactgtcaacccaggccttttgtattcattct

gcagctagaacttgggtgggtcttcatgcttgacaggcaagcaatttatggactaagctgttccctcggccctct

cttgacccatttaccagaaaggggggttccttgatcaatggcgaagccaggctggtgttcccaagaaagccttgac

tctgggtacagtgacctcagtggggtgagaggagttctcccccctagctgggctggggcccagctccacccccctca

ggctattcaatgggggtgcttccaggaagtcaggggcagatttagtccaacccgttcctccataaaggccctgac

atcccaggagccagcagaggcagggcacc

Supplementary Figure S5. The scheme of GFAP promoter with putative Nkx2.1 binding site and primers used for ChIP assay.

A. GFAP promoter sequence is shown with putative Nkx2.1 binding site in bold red font at position –838 bp relative to the putative transcriptional start site (shown in uppercase). The forward and reverse primers 5’- tggataagaggccacagagg and 5’-cctctcccctgaatctctcc that were used for ChIP assay are underlined.

B. GFAP promoter sequence from the *pDRIVE-mGFAP-LacZ* plasmid used for transfection experiments shown in Figure 9d-i. The putative Nkx2.1 binding site is shown in bold red font.
